# Stroke rehabilitation: from diagnosis to therapy

**DOI:** 10.3389/fneur.2024.1402729

**Published:** 2024-08-13

**Authors:** Xiaohong Li, Yanjin He, Dawu Wang, Mohammad J. Rezaei

**Affiliations:** ^1^Department of Rehabilitation Medicine, The First Affiliated Hospital of Chongqing Medical University, Chongqing, China; ^2^School of Medicine, Tehran University of Medical Sciences, Tehran, Iran

**Keywords:** stroke, rehabilitation, neuroplasticity, neurostimulation, motor learning

## Abstract

Stroke remains a significant global health burden, necessitating comprehensive and innovative approaches in rehabilitation to optimize recovery outcomes. This paper provides a thorough exploration of rehabilitation strategies in stroke management, focusing on diagnostic methods, acute management, and diverse modalities encompassing physical, occupational, speech, and cognitive therapies. Emphasizing the importance of early identification of rehabilitation needs and leveraging technological advancements, including neurostimulation techniques and assistive technologies, this manuscript highlights the challenges and opportunities in stroke rehabilitation. Additionally, it discusses future directions, such as personalized rehabilitation approaches, neuroplasticity concepts, and advancements in assistive technologies, which hold promise in reshaping the landscape of stroke rehabilitation. By delineating these multifaceted aspects, this manuscript aims to provide insights and directions for optimizing stroke rehabilitation practices and enhancing the quality of life for stroke survivors.

## 1 Introduction

Stroke stands as a significant global burden, impacting millions of lives annually and presenting a substantial challenge to healthcare systems worldwide. Statistics from the World Health Organization (WHO) reveal that stroke is a leading cause of mortality and disability globally. Annually, approximately 13.7 million new cases of stroke are reported, making it a prevalent health issue across continents and demographics (1). The latest findings from the 2019 Global Burden of Disease (GBD) report revealed that stroke continues to hold its position as the second leading cause of mortality and the third leading cause of mortality and disability combined, measured in terms of disability-adjusted life-years lost (DALYs) worldwide (2). The projected economic impact of stroke globally exceeds US$891 billion, accounting for approximately 1.12% of the global Gross Domestic Product (GDP) (3). Between 1990 and 2019, there was a significant surge in the burden of stroke, indicated by a 70.0% rise in new stroke cases, a 43.0% increase in stroke-related deaths, a 102.0% uptick in existing stroke cases, and a 143.0% elevation in DALYs. The majority of the worldwide burden of stroke, accounting for 86.0% of deaths and 89.0% of DALYs, was observed in countries categorized as lower-income and lower-middle-income countries (LMIC).

Considerable variations were evident in age-standardized stroke incidence (six-fold), mortality (15-fold), prevalence (four-fold), and DALYs (20-fold) rates across different geographical regions. The regions with the highest rates were predominantly observed in LMIC, notably in Eastern Europe, Asia, and Sub-Saharan Africa (4). Beyond its high incidence, stroke accounts for a substantial number of deaths globally. Those who survive often experience varying degrees of disability, which significantly affects their quality of life (5). Disabilities resulting from stroke encompass motor impairments, cognitive deficits, speech and language difficulties, and psychological challenges (6). The aftermath of stroke extends beyond the individual affected, impacting families, caregivers, and society at large. The disabilities and long-term care needs of stroke survivors impose emotional, financial, and practical burdens on families (7, 8). Furthermore, the societal cost is immense, encompassing healthcare expenses, loss of productivity, and the need for long-term rehabilitation and support services (9). Disparities in stroke incidence, management, and outcomes exist globally, often correlated with socio-economic factors (10). Low-and middle-income countries face greater challenges due to limited access to healthcare services, diagnostics, and rehabilitation facilities. These disparities contribute to higher mortality rates and poorer recovery outcomes in certain regions (11).

Efforts to reduce the global burden of stroke emphasize prevention through awareness campaigns targeting risk factors like hypertension, smoking, obesity, and physical inactivity (3). Additionally, interventions focusing on timely access to acute care, advanced treatments such as thrombolytic therapy, and comprehensive rehabilitation are crucial in mitigating the impact of stroke (12). Addressing the global burden of stroke necessitates collaborative efforts between governments, healthcare organizations, advocacy groups, and communities. Initiatives aimed at improving access to healthcare, enhancing stroke awareness, promoting healthier lifestyles, and advancing rehabilitation services are fundamental in reducing the burden of stroke on a global scale (13). The pervasive impact of stroke underscores the urgent need for concerted global action. Strategies encompassing prevention, timely intervention, access to quality healthcare, and comprehensive rehabilitation are essential to alleviate the burden of stroke and improve outcomes for individuals affected by this debilitating condition (14). This paper aims to provide a comprehensive overview of the evolving landscape of stroke rehabilitation, emphasizing the critical phases from diagnosis to therapy. By synthesizing current knowledge and highlighting emerging trends, this review intends to serve as a resource for clinicians, researchers, and policymakers involved in stroke care and rehabilitation. This manuscript endeavors to contribute to the evolving discourse on stroke rehabilitation by synthesizing and analyzing diverse literature from across the spectrum of stroke care. While not a systematic review in the traditional sense, this manuscript adopts a rigorous approach to literature selection, guided by clear inclusion criteria. These criteria prioritize relevance, quality, credibility, diversity of perspectives, and a keen focus on emerging technologies and innovations in stroke rehabilitation. Central to our approach is the recognition of the multifaceted nature of stroke rehabilitation. Each section of this manuscript delves into specific aspects of stroke care, from the early diagnosis and assessment to the implementation of rehabilitation programs across different stages of stroke recovery. By integrating evidence from various publication types, including original research articles, clinical trials, systematic reviews, meta-analyses, and review articles, we aim to provide a comprehensive overview of the current state of stroke rehabilitation. Moreover, our commitment to inclusivity extends beyond the traditional boundaries of stroke research. Efforts have been made to incorporate literature from diverse geographical regions, healthcare settings, and patient populations, acknowledging the unique challenges and perspectives that shape stroke rehabilitation practices globally.

Special attention is also given to the exploration of emerging technologies and innovations in stroke rehabilitation. From robotics to virtual reality and neurostimulation techniques, the evolving landscape of rehabilitation technology offers promising avenues for enhancing outcomes and improving the quality of life for stroke survivors. By embarking on this comprehensive review journey, we seek to not only consolidate existing knowledge but also identify gaps, challenges, and opportunities for future research and practice in stroke rehabilitation. Through our collective efforts, we aspire to contribute to the advancement of evidence-based, patient-centered care that optimizes outcomes and fosters resilience in the face of stroke.

The multifaceted field of stroke rehabilitation is explored in this review paper, providing a comprehensive overview of current practices, challenges, and future directions. The paper begins with a background on stroke rehabilitation, discussing its significance, historical context, and the evolution of practices. This section sets the stage for understanding the critical role of rehabilitation in stroke recovery. Next, current rehabilitation practices are examined, detailing interventions such as early rehabilitation strategies, physical therapy, occupational therapy, speech and language therapy, and cognitive rehabilitation. Each subsection highlights evidence-based practices and their impact on recovery. The challenges and opportunities in stroke rehabilitation are then addressed. Issues such as the lack of standardized timing for rehabilitation initiation, disparities in access to services, stroke-related infections, the need for individualized rehabilitation plans, and the importance of interdisciplinary collaboration are analyzed. Potential improvements and innovations are discussed. Finally, the future directions are explored. Emerging trends and advancements, including personalized rehabilitation approaches, the integration of neuroplasticity concepts, and the development of assistive technologies, are highlighted. These innovations are shown to have the potential to transform rehabilitation practices and improve outcomes for stroke survivors. This structured approach provides a clear roadmap for readers, ensuring a thorough coverage of each aspect of stroke rehabilitation. It offers valuable insights for clinicians, researchers, and stakeholders in the field.

## 2 Diagnosis of stroke: clinical assessment and imaging techniques

**Figure 1:**
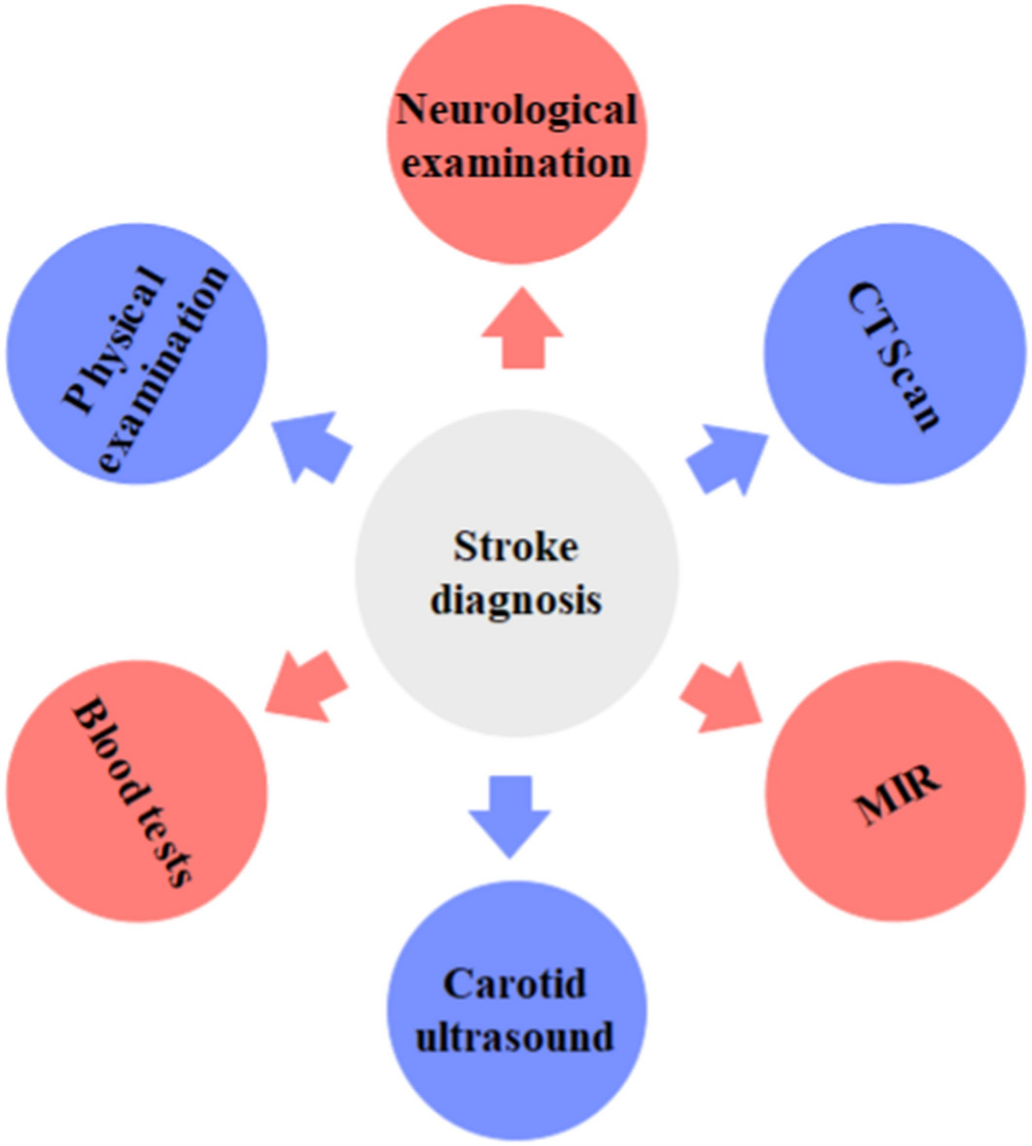
The most reliable test for clinical assessment of stroke.

The clinical assessment of stroke involves a comprehensive evaluation that begins with taking the patient’s medical history (Figure 1). This includes probing into risk factors such as hypertension, diabetes, smoking, previous strokes, or heart disease (15). A thorough physical examination follows, focusing on neurological assessments. This examination evaluates motor function, sensation, coordination, reflexes, and cranial nerve function (16). Utilizing scales like the NIH Stroke Scale (NIHSS) helps in quantifying the severity of stroke symptoms, aiding in treatment decisions (17). Additionally, determining the time of symptom onset is critical, as certain treatments like thrombolytic therapy have a limited window of effectiveness after symptom onset (18). Imaging serves as a pivotal component in selecting patients for intravenous and intra-arterial arterial ischemic stroke treatments. Techniques like computed tomography (CT) with CT angiography or magnetic resonance (MR) with MR angiography are employed to eliminate stroke mimics and hemorrhages, ascertain the stroke’s cause and mechanism, delineate the extent of brain infarction, and pinpoint arterial blockages (19). Imaging has the potential to discern patients who would derive greater benefit from revascularization therapies, irrespective of the traditional therapeutic time frame. This capability allows for personalized treatment decisions, enhancing individual patient outcomes significantly (20). Multiparametric CT/MR imaging can determine the scope of potentially salvageable brain tissue (penumbra) and irreversible brain damage (core) by leveraging CT perfusion and/or diffusion-weighted and perfusion-weighted MR imaging (21, 22). Additionally, the imaging of diffusion-weighted techniques aids in evaluating the status of arterial collateral circulation and discerning the type and spread of the clot (23).

The primary goals of arterial ischemic stroke imaging involve excluding hemorrhage and stroke mimics and identifying optimal candidates for IV or IA treatments, considering both the extent of confirmed brain damage (core) and the location of arterial blockages (24, 25). Arterial ischemic strokes are urgent medical situations where treatment decisions (ranging from conservative approaches to IV thrombolysis and/or mechanical revascularization) hinge on two crucial imaging factors: the timeframe since onset and two primary features observed in imaging—parenchymal lesions and arterial blockage locations (26, 27). Imaging of the parenchyma is vital for confirming the diagnosis and gauging the extent of ischemic damage, while vascular imaging, conducted through CT/CTA or MRI/MRA, is essential for pinpointing the location of arterial blockages in arterial ischemic stroke evaluations (28, 29). Supplementary details about collateral flow, penumbra, and core extension can significantly enhance the precision of individual treatment decisions. The selection of the initial imaging modality for arterial ischemic stroke assessment is largely influenced by the immediate 24/7 equipment accessibility and its capability to furnish vital information necessary for various treatment approaches (29, 30). Due to its broader availability, CT stands as the most widely utilized imaging technique globally for acute stroke. Transcranial Doppler (TCD) utilizes ultrasound to assess blood flow in the brain’s blood vessels, aiding in the evaluation of stroke and identifying conditions like vasospasm, providing valuable insights into the vascular aspects of stroke pathology (31). Telemedicine and mobile imaging have revolutionized stroke care, particularly in areas with limited access to specialized stroke expertise.

Remote evaluation of stroke patients using telemedicine enables timely assessment and consultation with stroke specialists, facilitating rapid decision-making and treatment initiation (32). Artificial intelligence (AI) is increasingly being integrated into imaging analysis, showing promising results in quickly and accurately detecting stroke. AI-assisted analysis helps clinicians by providing rapid insights into imaging data, aiding in prompt diagnosis and subsequent treatment planning (33). Emerging research highlights the capacity of AI to enhance stroke prognosis. Lukić et al. (34) pioneered an artificial neural network (ANN)-driven framework for predicting outcomes in cases of stroke. Meanwhile, Wang et al. (35) utilized machine learning to construct a random forest model for stroke prognosis. Additionally, Xu et al. (36) amalgamated CT radiomics with machine learning methods to devise stroke prognostic models integrating 18 CT imaging attributes. These novel approaches have optimized the selection of risk factors and expanded the inclusion of imaging features, thus bolstering predictive precision. Moreover, deep learning has surfaced as a hopeful avenue for prognostic forecasting leveraging imaging data. Chen et al. (37) formulated a predictive model centered on convolutional neural networks (CNNs), blending clinical data with extensive imaging characteristics to address prior constraints of stroke prognostic frameworks. Their results indicate heightened predictive efficacy of the clinic-imaging fusion CNN model. This model stands poised for integration into clinical practice, offering the prospect of enhanced management for ICH patients. These technological advancements in stroke diagnosis significantly improve the speed and accuracy of assessments, allowing for rapid initiation of appropriate treatments, thereby minimizing brain damage and enhancing the prospects of recovery for stroke patients.

## 3 Early identification of rehabilitation needs

Upon a patient’s arrival following a stroke, a comprehensive clinical assessment is crucial. This assessment involves a detailed evaluation of the patient’s neurological status, encompassing motor function, sensation, coordination, and cognitive abilities. It helps in swiftly identifying any deficits or impairments resulting from the stroke (38). Additionally, assessing speech, language, and swallowing abilities early on is vital. This immediate evaluation assists in identifying any communication impairments or dysphagia, indicating the need for specialized speech and language therapy (39).

Utilizing tools such as NIHSS aids in objectively quantifying the severity of stroke symptoms. This assessment provides critical information to healthcare professionals about the extent and severity of impairments, guiding decisions on the urgency and intensity of rehabilitation interventions (40). Functional assessments, like the Functional Independence Measure (FIM), play a crucial role in determining the patient’s level of independence in performing activities of daily living (ADLs). These assessments aid in highlighting specific areas requiring focused rehabilitation efforts (41). As mentioned previously, imaging techniques, such as CT scans MRI not only confirm the diagnosis of stroke but also offer insights into the location and extent of brain damage. Integrating these imaging findings with clinical deficits assists in understanding the specific deficits correlated with affected brain regions. This correlation is pivotal in guiding the development of targeted rehabilitation strategies tailored to the individual’s needs (21, 42).

Initiating rehabilitation interventions as early as possible post-stroke, even within the acute phase, is associated with improved outcomes. Early interventions may involve passive range-of-motion exercises, early mobilization, or cognitive stimulation exercises. These early initiatives aim to prevent secondary complications, promote neural plasticity, and lay the groundwork for subsequent rehabilitation efforts (43, 44). Concurrently, setting rehabilitation goals in collaboration with the patient and their caregivers helps establish a clear direction for therapy. These goals are individualized, realistic, and aimed at improving functional abilities based on the identified impairments (45). Involving a multidisciplinary team comprising various specialists—such as physiotherapists, occupational therapists, speech-language pathologists, psychologists, and social workers—is instrumental. This collaborative approach ensures a comprehensive assessment and identification of rehabilitation needs from various perspectives. Each specialist brings unique expertise to the table, contributing to the development of a holistic rehabilitation plan tailored to the patient’s specific impairments and goals (14). Furthermore, engaging family members or caregivers right from the beginning allows them to grasp the rehabilitation process, receive training on assisting the patient, and create a supportive environment upon the patient’s discharge (46). Early identification of stroke rehabilitation needs through a comprehensive and interdisciplinary approach sets the stage for a focused, individualized, and timely rehabilitation plan, significantly impacting the trajectory of recovery for stroke survivors (47).

## 4 Stages of stroke and corresponding rehabilitation programs

Stroke recovery is typically categorized into three stages: acute, subacute, and chronic. Each stage has distinct characteristics and rehabilitation needs, which are critical for optimizing patient outcomes (Table 1).

**Table 1:** Efficacy of various rehabilitation approaches across different stages of stroke recovery.

Type of rehabilitation	Efficacy	Stage of Stroke	References
Proprioceptive training	Improves balance and functional mobility; enhances sensory feedback, leading to better motor control and reduced fall risk	Subacute to chronic	(48–50)
Dual-task exercises	Enhances gait, balance, and cognitive function; promotes better motor-cognitive coordination and multitasking ability	Subacute to chronic	(51–54)
Goal-oriented activities	Improves functional outcomes and quality of life by setting specific, measurable goals tailored to the patient’s needs and capabilities	Acute to chronic	(55–58)
Constraint-induced movement therapy (CIMT)	Increases use of the affected limb through repetitive practice and restricting the unaffected limb, leading to improved motor function	Subacute to chronic	(59–62)
Robot-assisted therapy	Enhances upper limb motor recovery by providing consistent, high-intensity, and repetitive movements; improves precision and strength	Subacute to chronic	(63–66)
Virtual reality training	Improves motor function, engagement, and motivation through interactive and immersive environments; facilitates task-specific practice	Subacute to chronic	(56, 67–69)
Aerobic exercise	Enhances cardiovascular fitness, overall mobility, and endurance; contributes to better overall health and reduces risk of secondary complications	Subacute to chronic	(70–72)
Occupational therapy	Improves independence in ADLs by enhancing fine motor skills, cognitive abilities, and adapting environments	Acute to chronic	(73–75)
Speech and language therapy (SLT)	Enhances communication abilities, including speech, language, and swallowing functions; critical for improving social interactions and quality of life	Acute to chronic	(76–78)
Functional electrical stimulation (FES)	Improves muscle strength and motor control by applying electrical stimulation to paralyzed muscles, facilitating voluntary muscle contraction	Acute to chronic	(79–82)
Mirror therapy	Enhances motor recovery and reduces pain by creating a visual illusion of movement in the affected limb through mirror reflection	Subacute to chronic	(83–86)
Cognitive rehabilitation	Improves cognitive functions such as memory, attention, and problem-solving; essential for overall functional recovery and independence	Subacute to chronic	(87–90)
Hydrotherapy	Utilizes water resistance and buoyancy to improve strength, balance, and coordination; reduces pain and facilitates movement	Subacute to chronic	(91, 92)
Music therapy	Enhances mood, motivation, and motor function through rhythmic and musical cues; supports emotional well-being and recovery	Acute to chronic	(93–95)
Psychological counseling and support	Addresses emotional and mental health issues, including depression and anxiety, which are common post-stroke; improves overall well-being	Acute to chronic	(96–98)

### 4.1 Acute stage of stroke

The acute stage of stroke encompasses the first 24 h to 1 week following the onset of the stroke. During this initial phase, the primary focus is on medical stabilization and preventing further brain damage (99). Immediate medical interventions are crucial, including measures to restore blood flow, such as the administration of tissue plasminogen activator (tPA) for ischemic strokes or surgical procedures to address hemorrhagic strokes (100). Early rehabilitation efforts aim to prevent complications, such as deep vein thrombosis, pressure sores, and contractures. Gentle mobilization, proper positioning, and passive range of motion exercises are implemented to maintain some level of physical function. Basic activities of daily living (ADL) training, including assistance with eating, dressing, and personal hygiene, are introduced to help patients maintain a degree of independence. Psychological support is also essential at this stage to help patients and their families cope with the emotional impact of the stroke (101).

### 4.2 Subacute stage of stroke

The subacute stage occurs from 1 week to three to 6 months post-stroke. This phase is marked by more intensive rehabilitation efforts aimed at promoting neurological recovery and improving functional abilities (102). During the subacute stage, patients typically engage in comprehensive rehabilitation programs that include physical therapy, occupational therapy, and speech and language therapy. Physical therapy focuses on improving strength, coordination, balance, and mobility through exercises and activities tailored to the patient’s needs (103). Occupational therapy helps patients regain the ability to perform daily tasks, such as dressing, bathing, and cooking, by using techniques like constraint-induced movement therapy (CIMT) and dual-task training (104). Proprioceptive training is also incorporated to enhance the patient’s sense of body position and movement (105). Speech and language therapy continues to address communication and swallowing difficulties (106). Psychological counseling and support remain vital to address any emotional and cognitive challenges that arise during this phase of recovery (107).

### 4.3 Chronic stage of stroke

The chronic stage of stroke begins 6 months after the initial event and extends indefinitely. During this phase, the focus shifts to long-term rehabilitation and the management of any residual disabilities. The goal is to maintain and further improve functional abilities while helping patients adapt to their long-term needs (55). Rehabilitation interventions in the chronic stage often include community-based programs, which provide ongoing therapy and support. Advanced therapies such as robot-assisted therapy and virtual reality training are used to enhance motor skills and cognitive functions (108). Functional electrical stimulation (FES) may be employed to improve muscle function, and mirror therapy can be used to create the illusion of movement in the affected limb (109). Cognitive rehabilitation activities are designed to improve memory, attention, and problem-solving skills. Aerobic exercises and hydrotherapy are included to improve cardiovascular health and overall fitness (70). Music therapy can be beneficial for enhancing mood and cognitive functions (110). Long-term psychological support is crucial for helping patients adjust to their new normal and maintain motivation for continued recovery (111).

## 5 Acute management and rehabilitation

In the critical phase immediately following a stroke, the imperative is to promptly implement rehabilitation initiatives tailored to address immediate needs and initiate the recovery process. This phase is characterized by a dual focus on immediate rehabilitation strategies and addressing acute medical requirements. Acute management in stroke rehabilitation refers to the immediate interventions and care provided after the initial medical stabilization of a stroke patient, typically within the first few days to weeks following the stroke. This phase is distinct from emergency medical care and focuses on beginning the rehabilitation process to optimize recovery and prevent complications (40). After the initial emergency treatment, which aims to stabilize the patient and address life-threatening issues, acute management begins to initiate the rehabilitation journey. The goal during this phase is to start therapies that can enhance neurological recovery, improve functional outcomes, and set the stage for long-term rehabilitation (112). This involves a multidisciplinary approach, including neurologists, physical therapists, occupational therapists, speech therapists, and other healthcare professionals working together to develop a comprehensive care plan (101).

Continuous monitoring of neurological status and vital signs is crucial for detecting any signs of deterioration or complications such as recurrent strokes, infections, or cardiac issues. Adjustments to medication regimens and treatments are made based on the patient’s evolving condition (113). Initiating gentle physical activities and mobilization as soon as the patient is medically stable is essential. Early mobilization helps prevent complications like deep vein thrombosis, muscle atrophy, and joint contractures. Physical therapists work with patients to perform passive and active range of motion exercises, bed mobility exercises, and, when possible, supported standing and walking activities (114). Occupational therapists focus on helping patients regain their ability to perform daily activities. This includes exercises and interventions designed to improve motor skills, coordination, and cognitive functions necessary for tasks such as dressing, eating, and personal hygiene (115). For patients experiencing aphasia or dysphagia, speech therapists conduct assessments and provide targeted therapies to improve communication and swallowing abilities. This is critical to ensure that patients can safely consume food and liquids, thus reducing the risk of aspiration pneumonia (116). Addressing the psychological impact of a stroke is a key component of acute management. Mental health professionals provide counseling and support to help patients and their families cope with the emotional aftermath of a stroke. This support can help in reducing anxiety and depression, fostering a positive outlook towards rehabilitation (14). Proactive measures are taken to prevent secondary complications, including managing risk factors for recurrent strokes, such as hypertension and diabetes, ensuring proper nutrition, and maintaining skin integrity to prevent pressure ulcers (117). Thus, The primary objectives of acute management in stroke rehabilitation are to stabilize the patient’s medical condition, initiate rehabilitation therapies to enhance recovery, prevent secondary complications such as infections, thromboembolic events, and muscle wasting, provide psychological support to help patients and families adjust to the changes brought by the stroke, and establish a foundation for the subsequent stages of rehabilitation. By addressing these aspects, acute management aims to maximize the potential for functional recovery and improve the overall quality of life for stroke patients in the critical early days following their stroke.

### 5.1 Early rehabilitation initiatives

In the crucial hours immediately after a stroke, the introduction of early rehabilitation initiatives holds profound significance. These pivotal interventions, instated within the initial 24 to 48 h following the stroke event, form the cornerstone of the recovery process (118). Their primary objectives encompass preventing potential complications, initiating the rehabilitation trajectory, and laying the essential groundwork for optimal recovery (119).

Passive range-of-motion exercises emerge as fundamental components even during the acute phase post-stroke. These gentle movements administered to the affected limbs serve as a safeguard, preserving joint flexibility and thwarting the onset of muscle contractures. This preventive measure is instrumental in mitigating the risks associated with long-term impairments that could impede the recovery process (120, 121). Early mobilization takes precedence as a pivotal strategy in acute rehabilitation. Encouraging and facilitating patients to engage in sitting exercises, standing movements, and, when feasible, undertaking short walks constitute early mobilization efforts (71). Beyond mitigating muscle deconditioning, these early mobilization initiatives significantly mitigate the likelihood of complications like deep vein thrombosis and pressure ulcers, essential for promoting a conducive environment for recovery (122, 123). Sensory stimulation techniques, involving tactile, auditory, or visual stimulation, are employed to arouse and activate affected senses. This early engagement of the senses aims to jumpstart neural plasticity and sensory reawakening in the specific brain regions impacted by the stroke event (124, 125). While seemingly straightforward, these early rehabilitation initiatives wield a profound impact. Their implementation is consistently validated by research, underscoring their pivotal role in shaping the trajectory of recovery for stroke survivors.

### 5.2 Addressing mobility and functional impairments

**Figure 2:**
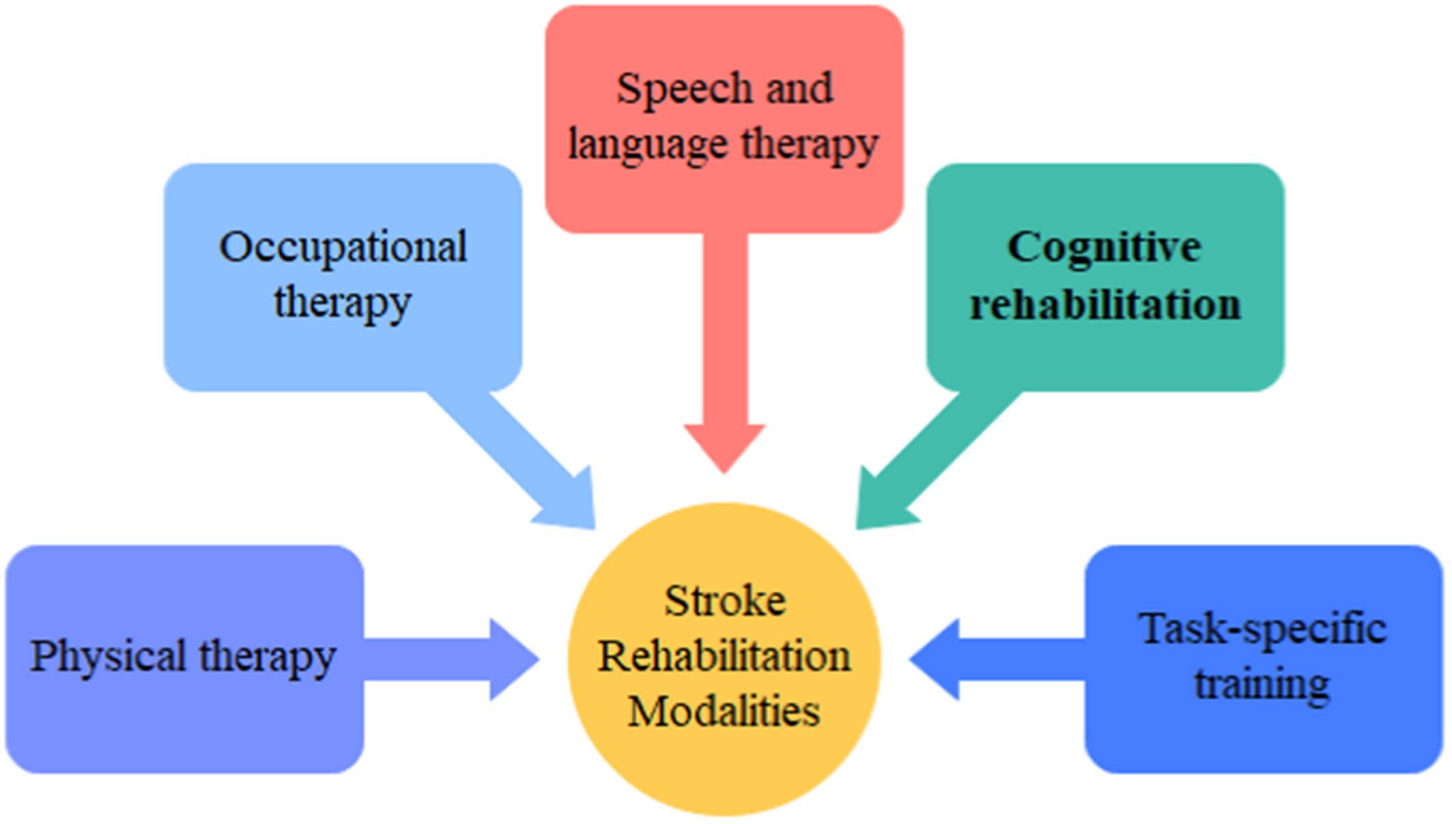
Different stroke rehabilitation modalities.

In the acute phase of stroke rehabilitation, the focus sharply narrows to addressing mobility and functional impairments. This critical phase aims to reestablish lost capabilities, enhance balance, coordination, and restore independence in daily activities (Figure 2). Physiotherapy interventions form the backbone of efforts to regain mobility. Gait training, strength-building exercises, and balance drills orchestrated by physiotherapists are instrumental in improving motor function. These specialized exercises and techniques, often personalized to the individual’s condition, actively work toward ameliorating gait abnormalities. The integration of assistive devices and adaptive strategies further facilitates safe and effective movement (126, 127). Occupational therapy takes center stage in reinstating functionality in ADLs. Occupational therapists guide patients through essential tasks, such as dressing, grooming, feeding, and home management. The focus lies in enabling patients to regain independence in these pivotal daily activities, promoting autonomy and self-sufficiency (128). Task-specific training, an integral component of acute rehabilitation, involves engaging patients in activities tailored precisely to their deficits. By replicating real-life situations, this method fosters neural reorganization and functional recovery. These activities, meticulously designed to confront the unique challenges posed by the stroke, play a pivotal role in retraining the brain and restoring lost functionalities (56, 129). This acute phase of rehabilitation, deeply rooted in tailored interventions and guided exercises, serves as the foundation upon which subsequent recovery efforts are built. By addressing mobility and functional impairments strategically, this phase contributes significantly to restoring independence and enhancing the quality of life for stroke survivors (87).

### 5.3 Integrating rehabilitation into acute care

Integrating rehabilitation seamlessly into acute care settings is a fundamental aspect of providing comprehensive stroke care. This integration involves merging rehabilitation strategies into the acute medical management plan, ensuring a cohesive approach to address immediate medical needs alongside rehabilitation goals (130). Early identification and team collaboration are pivotal in this integration. Identifying rehabilitation needs promptly and assembling a multidisciplinary team comprising neurologists, physiatrists, nurses, therapists, and social workers facilitates a comprehensive assessment. This collaborative effort allows for a simultaneous evaluation of rehabilitation requirements in tandem with acute medical interventions (131). Tailoring rehabilitation goals forms the cornerstone of the integrated approach. Setting specific, measurable, achievable, relevant, and time-bound (SMART) objectives is crucial. These objectives, personalized to the patient’s condition, aim to optimize functional recovery while concurrently addressing immediate medical concerns. This tailored framework for rehabilitation forms the bedrock of a responsive and dynamic rehabilitation plan (132). Continuous monitoring and adjustment of rehabilitation strategies are essential components of this integrated model. Continuous evaluation of the patient’s response to rehabilitation interventions enables real-time adjustments. This adaptive approach ensures that rehabilitation strategies remain responsive to the patient’s evolving needs and medical condition, fostering a flexible and patient-centric rehabilitation process (133, 134). Integrating rehabilitation into acute care represents a paradigm shift, acknowledging the symbiotic relationship between immediate medical interventions and the imperative need for rehabilitative efforts. This holistic approach not only kickstarts the rehabilitation journey early on but also ensures a coordinated and comprehensive care plan for stroke survivors (19, 135).

## 6 Rehabilitation modalities

### 6.1 Physical therapy

Physical therapy (PT) stands as a cornerstone within stroke rehabilitation, encompassing a multifaceted approach aimed at restoring mobility, enhancing motor function, and mitigating physical impairments. It serves as an integral component throughout the continuum of stroke care, spanning from acute management to long-term recovery (136, 137). Upon initial assessment, physical therapists meticulously evaluate the individual’s functional abilities, motor impairments, and specific limitations resulting from the stroke. Based on this assessment, personalized rehabilitation plans are crafted, and tailored to address the unique needs and goals of each patient. These plans are dynamic, evolving with the patient’s progress and changing requirements (138, 139). Gait training assumes a crucial role in PT for stroke survivors, focusing on restoring and optimizing walking patterns.

Therapists employ various techniques, from treadmill-based training to over-ground walking practice, concentrating on improving balance, strength, and coordination (140). Advanced technologies such as robotic-assisted gait training or body-weight-supported treadmill training may be utilized to augment the rehabilitation process. Emphasis is placed on re-establishing a safe and functional gait to enable increased independence in daily activities (141). PT interventions encompass a range of exercises designed to enhance muscle strength, flexibility, and coordination. Targeted exercises, customized to address specific muscle groups affected by the stroke, aid in motor recovery. Therapists utilize resistance training, functional activities, and neuromuscular re-education to promote muscle activation and facilitate movement in impaired limbs (142, 143). Therapists employ various balance exercises and proprioceptive activities to improve balance and coordination, essential for preventing falls and enabling functional mobility. These exercises often involve challenging the patient’s stability in a controlled environment to enhance balance reactions and postural control (144). Moreover, to mitigate the risk of secondary complications such as joint contractures and stiffness, therapists incorporate range-of-motion exercises. These gentle movements aim to preserve joint flexibility, prevent musculoskeletal issues, and facilitate ease of movement in affected limbs (145, 146). PT extends beyond clinical settings, encompassing functional training and home modification recommendations. Therapists collaborate with patients to simulate real-life scenarios, practicing activities crucial for daily living. Additionally, they provide guidance on adapting home environments and suggesting assistive devices and modifications to enhance accessibility and safety within the home setting (147, 148). Physical Therapy, as a core element of stroke rehabilitation, not only targets physical impairments but also promotes functional independence, enabling individuals to regain confidence and improve their overall quality of life following a stroke.

### 6.2 Occupational therapy

Occupational therapy (OT) within stroke rehabilitation is a pivotal modality focused on restoring independence in daily activities, re-establishing functional abilities, and promoting engagement in meaningful life roles. Tailored to each individual’s needs and goals, OT interventions span various domains to facilitate a smoother transition from impairment to autonomy (149, 150). Occupational therapists conduct comprehensive assessments to gauge the impact of stroke on an individual’s ability to perform daily tasks. These assessments help formulate personalized rehabilitation plans, targeting specific deficits in ADLs, instrumental activities of daily living (IADLs), work-related tasks, and leisure activities (151, 152). OT interventions primarily revolve around retraining and adapting ADLs. Therapists employ task-oriented training to facilitate independence in self-care activities such as grooming, bathing, dressing, toileting, and feeding. Adaptive strategies, environmental modifications, and the use of assistive devices are integrated to maximize functional independence and participation in daily routines (153, 154). Recovery of upper limb function is a key focus within OT. Therapists utilize exercises targeting hand-eye coordination, fine motor control, and dexterity. Techniques like constraint-induced movement therapy (CIMT), mirror therapy, and graded motor imagery are employed to encourage the use of the affected limb and improve its function (155). OT extends its scope to support community reintegration and vocational pursuits. Therapists assist individuals in transitioning back into community life by providing guidance on accessing community resources, public transportation, and social participation. Vocational rehabilitation focuses on adapting work environments, modifying job tasks, and exploring adaptive technologies to facilitate a return to work or engagement in meaningful productive activities (156, 157).

Occupational therapists address cognitive impairments and sensory deficits that impact daily functioning. Strategies are implemented to improve attention, memory, executive functions, and problem-solving skills necessary for effective task performance. Sensory integration techniques help individuals process sensory information effectively, enhancing their ability to engage in meaningful activities (158). Collaborating with individuals and their families, OTs recommend modifications in the home environment to promote safety, accessibility, and independence. Suggestions for adaptive equipment and assistive devices are provided to facilitate ease in performing tasks and to compensate for functional limitations (159). OT, as an integral part of stroke rehabilitation, focuses on enabling individuals to regain independence and participation in activities essential to their daily lives, fostering a sense of accomplishment and self-sufficiency post-stroke.

### 6.3 Speech and language therapy

Speech and language therapy (SLT) is a critical component of stroke rehabilitation, encompassing a diverse range of interventions tailored to address communication and swallowing difficulties. These interventions aim to restore functional communication abilities and ensure safe swallowing, significantly impacting an individual’s quality of life post-stroke (39). Speech-language pathologists conduct comprehensive assessments to evaluate the extent of communication impairments and swallowing difficulties post-stroke. These assessments form the basis for individualized treatment plans that address specific deficits and goals (160). SLT interventions for speech articulation aim to improve clarity, intelligibility, and pronunciation. Therapists utilize exercises targeting oral-motor coordination, breath control, and vocal exercises to enhance speech production (161). Techniques such as articulation drills, repetition exercises, and tongue-strengthening exercises aid in improving speech intelligibility (162). Language therapy focuses on improving language comprehension and expression abilities. Therapists employ activities like word-finding exercises, storytelling, and comprehension tasks to enhance language skills. Strategies aim to rebuild vocabulary, sentence construction, and overall language fluency (163).

SLT addresses cognitive-communication deficits resulting from stroke, including impairments in problem-solving, reasoning, and social communication skills. Therapists use tasks such as conversation practice, problem-solving scenarios, and pragmatic language exercises to improve overall communication abilities (164). Swallowing difficulties, known as dysphagia, are common post-stroke. SLT interventions target these issues to ensure safe and efficient swallowing. Therapists employ various exercises to improve muscle coordination and swallowing reflexes (165). Strategies like modified diets, swallowing maneuvers, and postural adjustments are introduced to minimize the risk of aspiration and enhance swallowing function (166). For individuals with severe communication impairments, SLT involves introducing augmentative and alternative communication (AAC) strategies. These may include communication boards, speech-generating devices, or apps that enable individuals to express themselves effectively, compensating for speech difficulties (167). SLT involves educating families and caregivers about communication strategies and techniques to facilitate effective communication with the individual post-stroke. Training in supportive communication approaches fosters better understanding and interaction within the home environment (168). SLT, as a crucial element in stroke rehabilitation, aims to restore communication abilities and ensure safe swallowing, empowering individuals to engage more fully in social interactions and daily activities. A systematic review and meta-analysis by Chiaramonte and Vecchio (169) demonstrated the efficacy of speech rehabilitation in treating dysarthria among stroke survivors. The study observed notable enhancements in essential acoustic parameters, including alternating and sequential motion rates (AMR and SMR) and maximum phonation time, post-speech therapy. These parameters are vital for evaluating and managing dysarthria as they are directly linked to motor control necessary for speech production. Utilizing tools such as the Multi-Dimensional Voice Program (MDVP) and PRAAT software for acoustic analysis enables an objective measurement of improvements in patients’ speech intelligibility and supports targeted therapeutic interventions. Notably, techniques that increase jaw movement, promote louder speech, and expand vowel space area have been found to significantly enhance speech clarity and intelligibility. Incorporating these detailed assessments and targeted interventions into SLT programs can lead to more effective rehabilitation outcomes, thereby improving communication abilities and overall quality of life for stroke survivors (169).

### 6.4 Cognitive rehabilitation

Cognitive rehabilitation plays a vital role in stroke recovery, focusing on addressing cognitive impairments such as memory deficits, attention issues, and executive function difficulties. This multifaceted approach aims to restore and enhance cognitive abilities, ultimately promoting independence and improving the individual’s quality of life post-stroke (170). Cognitive rehabilitation begins with a comprehensive assessment of cognitive functions post-stroke. Therapists evaluate memory, attention, problem-solving, and executive function to identify specific deficits. Based on this assessment, tailored interventions are designed to address individual needs (88). Therapists employ various memory-enhancing techniques to improve memory recall and retention. These strategies may include mnemonic techniques, spaced-repetition exercises, memory games, and the use of memory aids like calendars or notebooks. By practicing memory recall, individuals can strengthen their ability to retain and retrieve information (171, 172). Interventions targeting attention deficits involve exercises that aim to improve different aspects of attention, including sustained attention, selective attention, and divided attention (173). Therapists utilize tasks requiring sustained focus, attentional drills, and multitasking activities to enhance attentional abilities and reduce distractibility (174). Cognitive rehabilitation addresses deficits in executive functions such as planning, problem-solving, and decision-making. Therapists employ tasks that require organization, time management, and prioritization to enhance cognitive flexibility and executive control. Strategies focus on breaking tasks into manageable steps and implementing problem-solving techniques (175). For individuals experiencing cognitive-communication deficits, therapists introduce strategies that enhance communication effectiveness. Techniques may involve using visual aids, simplifying information, and employing structured communication methods to aid comprehension and expression (176). Interventions extend to practicing functional tasks relevant to daily life. Therapists guide individuals through activities that replicate real-world scenarios, such as meal planning, managing finances, or using public transportation. Task-specific training helps individuals adapt to challenges they might encounter in their daily routines (177). Therapists collaborate with individuals to develop compensatory strategies and routines to cope with cognitive deficits. Additionally, recommendations for environmental modifications, such as organizing living spaces or implementing memory aids, assist individuals in managing daily tasks more effectively (178). Cognitive rehabilitation within stroke rehabilitation focuses on empowering individuals to adapt and compensate for cognitive impairments, enhancing their ability to perform daily activities independently and facilitating a smoother reintegration into their daily routines post-stroke.

### 6.5 Integration of proprioceptive training, dual-task exercises, and goal-oriented activities in stroke rehabilitation

Stroke rehabilitation aims to restore balance and autonomy in ADL through a variety of targeted exercises. Proprioceptive training, which focuses on improving body position awareness, is crucial for enhancing balance and coordination. Techniques such as balance boards, stability exercises, and sensory re-education are employed to stimulate proprioceptive feedback, promoting better motor control and reducing fall risk (105).

Dual-task exercises, which require patients to perform a motor task while simultaneously engaging in a cognitive task, have shown significant benefits in stroke recovery. These exercises not only improve physical function but also enhance cognitive processing, which is often impaired in stroke survivors. For instance, walking while counting backward or performing a physical task while responding to questions helps integrate cognitive and motor skills, leading to improved overall function (179, 180).

Goal-oriented exercises involve setting specific, measurable, achievable, relevant, and time-bound (SMART) goals that align with the patient’s individual needs and capabilities. These exercises foster motivation and adherence to the rehabilitation program (181). By focusing on personal goals, such as dressing independently or walking a certain distance, patients are more likely to stay engaged and committed to their recovery process. Research has shown that goal-setting significantly improves outcomes in stroke rehabilitation by providing clear targets and enhancing patient autonomy (182).

Incorporating proprioceptive training, dual-task exercises, and goal-oriented exercises into stroke rehabilitation programs offers a comprehensive approach to improving balance and autonomy in ADL. Proprioceptive training enhances sensory feedback and coordination, dual-task exercises improve multitasking abilities and overall functional performance, and goal-oriented exercises ensure that rehabilitation is relevant and motivating for the patient. Together, these interventions address both the physical and cognitive challenges faced by stroke survivors, leading to more effective rehabilitation outcomes. Future research should continue to explore the optimal strategies for integrating these approaches to maximize their benefits for stroke patients.

### 6.6 Botulinum toxin injection in post-stroke spasticity

Spasticity resulting from strokes poses considerable hurdles to motor function and daily tasks for those affected. Botulinum toxin type A (BoNT-A) injection has become the preferred treatment for localized spasticity, especially among stroke survivors, owing to its established effectiveness, reversible nature, and minimal risk of complications. The treatment of upper motor neuron syndrome, marked by spasticity and excessive muscle activity, requires strategies to alleviate spasticity-related issues and forestall further complications (183). BoNT-A treatment has become a crucial therapeutic approach, especially for localized spasticity in individuals who have had strokes. Research exploring the effectiveness of elevated doses of BoNT-A (exceeding usual guidelines) in post-stroke spasticity has shown encouraging outcomes (184). Despite initial worries about potential systemic side effects, most patients encountered substantial decreases in spasticity without encountering severe issues (185, 186). Although elevated doses of BoNT-A demonstrated effectiveness in spasticity reduction, instances of adverse effects, including contralateral weakness and systemic complications, were observed in certain cases (187). It is essential to carefully assess injection frequency, dilution volumes, and the possibility of antibody formation to minimize the likelihood of adverse events (188). The results highlight the efficacy of elevated doses of BoNT-A in managing post-stroke spasticity, especially in scenarios involving significant muscle hypertonia (189). Nonetheless, meticulous patient selection, accurate injection methodologies, and vigilant monitoring for adverse effects are crucial to enhancing treatment efficacy and upholding patient well-being. Research findings indicate the potential benefits of increased BoNT-A doses in mitigating spasticity and enhancing functional recovery among stroke survivors (190). Additional investigations are needed to clarify the most effective dosing regimens, improve injection methodologies, and assess the long-term safety and effectiveness in broader patient populations.

## 7 Technological advances in rehabilitation

Advancements in technology have revolutionized stroke rehabilitation, and one prominent innovation is the integration of robotics. Robotic-assisted rehabilitation systems offer a promising avenue to enhance motor recovery, functional outcomes, and patient engagement in stroke rehabilitation.

### 7.1 Robotics in stroke rehabilitation

Technological advancements, particularly in robotics, have dramatically transformed the landscape of stroke rehabilitation, ushering in innovative approaches to enhance motor recovery, functional abilities, and overall quality of life for individuals affected by stroke (191). In upper limb rehabilitation, robotics play a pivotal role by offering precise and intensive therapy through devices like exoskeletons and end-effector robots. These devices provide tailored exercises, guiding patients through movements aimed at improving motor control, strength, and the range of motion in affected arms and hands. This focused approach allows therapists to customize rehabilitation plans based on specific impairments, fostering more targeted and impactful interventions (192, 193). Gait training, crucial for restoring walking abilities post-stroke, benefits significantly from robotic systems. Exoskeletons and treadmill-based robots assist individuals by providing mechanical support and guidance during walking exercises (194). These devices help in relearning proper gait patterns, correcting asymmetries, and improving balance and stride length. The real-time feedback offered by these systems enhances motor learning, aiding in the retraining of gait patterns (195). One of the significant advantages of robotic-assisted therapy lies in its ability to offer task-specific training. Therapists can replicate real-life activities through these systems, allowing stroke survivors to practice movements required for daily tasks. This tailored approach fosters neuroplasticity, encouraging the brain to adapt and rewire neural connections to facilitate improved movement patterns (196).

Additionally, these robotic systems provide therapists with real-time feedback and monitoring capabilities. They track performance metrics such as range of motion, force exertion, and movement accuracy. This data empowers therapists to assess progress accurately, modify therapy intensity, and customize treatment plans according to individual responses, ensuring optimized rehabilitation outcomes (197). The advent of portable and user-friendly robotic devices has extended the reach of rehabilitation beyond clinical settings. Home-based rehabilitation using these systems enables stroke survivors to continue their therapy under professional guidance. This continuity in therapy enhances accessibility and adherence to treatment, contributing to sustained recovery (198). Furthermore, the integration of adaptive algorithms powered by machine learning and artificial intelligence tailors rehabilitation interventions to individual needs. These algorithms analyze patient responses in real-time, allowing for adjustments in therapy parameters to maximize the effectiveness of rehabilitation protocols (199).

The interactive nature of robotic technologies in stroke rehabilitation engages patients through gamified exercises and interactive interfaces. This enhanced engagement motivates individuals to actively participate in therapy sessions, promoting adherence and ultimately contributing to improved outcomes and functional recovery (200). However, it is essential to acknowledge certain limitations associated with the use of robotics in rehabilitation. While robotics offer precise and intensive therapy, some challenges need to be addressed. One such limitation is the cost involved in acquiring and maintaining robotic systems, which may pose financial barriers to widespread adoption. Moreover, in certain usage protocols, such as assistive modes, there is a risk of decreased effort or attention from patients during training sessions. This decrease in engagement could potentially impact the effectiveness of rehabilitation outcomes. Therefore, it is crucial for therapists to carefully monitor patient participation and motivation levels during robotic-assisted therapy to ensure optimal results (201–204). Despite these challenges, the benefits of robotic systems in stroke rehabilitation are considerable. Overall, while acknowledging the limitations, robotics in stroke rehabilitation represent a promising frontier in enhancing motor recovery and functional independence. These advancements pave the way for more personalized, engaging, and effective interventions, offering hope for improved rehabilitation outcomes for stroke survivors.

### 7.2 Virtual reality and augmented reality applications

Technological advancements in virtual reality (VR) and augmented reality (AR) have profoundly impacted the landscape of stroke rehabilitation, offering immersive, interactive, and personalized interventions that significantly contribute to the recovery process (205).

Within stroke rehabilitation, VR therapy creates computer-generated environments that simulate real-life scenarios. These environments immerse patients in interactive exercises tailored to their specific rehabilitation needs. VR-based tasks focus on improving motor functions, cognitive abilities, and balance (206). For instance, individuals can engage in upper limb exercises or practice balance-related activities within a controlled virtual setting. By replicating real-world scenarios, VR therapy stimulates neuroplasticity and motor learning, aiding in functional recovery (207).

Augmented reality (AR) enhances rehabilitation by overlaying digital information onto the real world. AR applications provide real-time visual cues or instructions during exercises, aiding stroke survivors in performing movements accurately and safely (208). For example, a stroke survivor practicing arm movements may receive visual guidance overlaid onto their affected limb, promoting correct positioning and motion execution. AR enhances spatial awareness and motor planning, facilitating better task performance (209).

In cognitive rehabilitation, VR and AR interventions extend beyond physical movements to address deficits in attention, memory, and executive function. Gamified tasks and simulations challenge cognitive skills, stimulating neural adaptation and functional improvement (210). These technologies offer engaging and interactive cognitive exercises, fostering mental stimulation and promoting cognitive recovery post-stroke (211). Moreover, VR and AR applications allow stroke survivors to simulate functional tasks in a controlled virtual environment. Patients can practice activities such as cooking, shopping, or navigating public spaces within the safety of a virtual setting. These simulations offer a platform for individuals to relearn and regain confidence in performing daily tasks, preparing them for real-life scenarios and promoting independence (212, 213).

However, it is crucial to recognize that the applicability of VR therapy may vary among stroke survivors. Factors such as cognitive abilities, physical impairments, and individual preferences need to be considered when determining the suitability of VR interventions for a particular patient. While VR therapy holds tremendous potential for many individuals, it may not be suitable for everyone. Therefore, a comprehensive assessment on a case-by-case basis is essential to ensure that VR therapy is appropriately tailored to meet the unique needs and capabilities of each stroke survivor (214, 215).

Despite the need for individualized assessment, VR and AR applications have demonstrated significant potential to enhance motivation and engagement during rehabilitation sessions. The immersive nature of these interventions captures individuals’ attention, encouraging active participation in therapy. The interactive and enjoyable aspects of VR and AR contribute to increased adherence and consistency in rehabilitation exercises, positively impacting recovery outcomes. Overall, while recognizing the transformative impact of VR and AR technologies on stroke rehabilitation, it is essential to assess their applicability on a case-by-case basis. By conducting individualized assessments and tailoring interventions to meet the unique needs of each patient, therapists can maximize the effectiveness of VR and AR applications in promoting motor, cognitive, and functional recovery for stroke survivors (56, 216, 217).

### 7.3 Neurostimulation techniques

Technological advancements in neurostimulation techniques have emerged as promising interventions within the realm of stroke rehabilitation, seeking to modulate neural activity, induce neuroplastic changes, and ultimately enhance recovery outcomes for individuals impacted by stroke.

#### 7.3.1 Transcranial magnetic stimulation

Transcranial magnetic stimulation (TMS) stands as a non-invasive neurostimulation technique used in stroke rehabilitation to modulate cortical excitability. It involves generating brief magnetic pulses through a coil placed over specific areas of the scalp, creating an electromagnetic field that penetrates the skull and influences neural activity in the underlying brain regions (218, 219). In stroke rehabilitation, repetitive TMS (rTMS) aims to induce changes in cortical excitability by delivering repeated magnetic pulses to targeted brain areas associated with motor function. By adjusting the frequency, intensity, and duration of these pulses, clinicians can modulate neural activity and promote neuroplasticity (220). The primary goal of TMS in stroke recovery is to facilitate motor relearning and functional improvement in affected limbs. By targeting specific cortical regions related to motor control and movement execution, rTMS aims to promote neural reorganization, facilitating the formation of new neural connections or enhancing existing ones. This process supports motor learning and reacquisition of motor skills affected by stroke-induced impairments (221, 222). Moreover, TMS is utilized to assess and map cortical excitability and the integrity of motor pathways in stroke survivors. By evaluating the response of motor cortices to TMS pulses, clinicians gain insights into the extent of cortical reorganization, helping to tailor and optimize rehabilitation strategies for individual patients (223). TMS holds promise as an adjunctive therapy in stroke rehabilitation, potentially enhancing outcomes by promoting neuroplastic changes, aiding in motor recovery, and improving functional abilities in affected individuals. Continued research and refinement of TMS protocols offer significant potential for optimizing its application in stroke rehabilitation, contributing to improved recovery and quality of life for stroke survivors.

#### 7.3.2 Transcranial direct current stimulation

Transcranial direct current stimulation (tDCS) is a non-invasive neurostimulation technique used in stroke rehabilitation to modulate cortical excitability. It involves delivering low-intensity electrical currents via electrodes placed on the scalp, aiming to influence neuronal activity in targeted brain areas (224). In the context of stroke recovery, tDCS seeks to promote neuroplastic changes by modulating cortical excitability in regions affected by the stroke-induced impairments (225). By applying a continuous low electrical current to specific cortical areas, tDCS aims to either enhance or inhibit neuronal firing patterns, ultimately influencing the brain’s ability to reorganize and adapt (226). The primary objective of tDCS in stroke rehabilitation is to facilitate neuroplasticity and enhance motor recovery. By modulating cortical excitability in motor-related brain regions, tDCS aims to promote the reorganization of neural networks, aiding in motor relearning and the restoration of motor function in affected limbs (227). Additionally, tDCS protocols can vary, employing different electrode montages and stimulation parameters to target specific cortical areas associated with motor function. The modulation of cortical excitability using tDCS may facilitate motor learning processes and the reacquisition of motor skills impacted by stroke-related impairments (228). Moreover, tDCS is considered a safe and well-tolerated technique, often used in conjunction with other rehabilitation approaches. Its non-invasive nature and potential to induce neuroplastic changes make it an attractive adjunctive therapy in stroke rehabilitation (229). Continued research into optimal tDCS parameters, such as electrode placement, stimulation duration, and intensity, holds promise for refining its application in stroke recovery. As the understanding of tDCS mechanisms grows, it offers substantial potential to enhance motor recovery outcomes and improve functional abilities in stroke survivors.

#### 7.3.3 Functional electrical stimulation

Functional electrical stimulation (FES) is a rehabilitative technique employed in stroke rehabilitation to activate paralyzed or weakened muscles by delivering controlled electrical impulses to peripheral nerves or muscle groups. It aims to restore voluntary movement, improve muscle strength, and facilitate motor relearning in affected limbs (229). In stroke survivors experiencing motor impairments, FES utilizes electrodes placed on the skin’s surface or implanted directly on muscles to deliver electrical stimulation (230). This stimulation generates muscle contractions, facilitating movements that may be difficult or impossible due to stroke-induced paralysis or weakness (231). The primary goal of FES in stroke rehabilitation is to support motor recovery and functional improvement. By stimulating targeted muscles or muscle groups, FES assists in muscle strengthening, preventing muscle atrophy, and enhancing muscle performance. This electrical stimulation aids in restoring voluntary movement patterns, facilitating motor control, and promoting the relearning of motor skills necessary for activities of daily living (232). FES applications can vary based on the specific rehabilitation goals and affected muscle groups. For instance, in upper limb rehabilitation, FES may target muscles involved in hand grasping or arm movement, aiding in functional tasks such as reaching or grasping objects. In lower limb rehabilitation, FES can assist with walking by stimulating leg muscles to facilitate stepping movements (233).

Furthermore, FES can be integrated into functional tasks or exercises, enabling stroke survivors to actively engage in movements with the assistance of electrical stimulation. This integration of FES with rehabilitation exercises promotes motor learning, supports task-specific training, and enhances functional recovery (234).

However, it is important to acknowledge that various studies have reported concerns regarding the discomfort associated with wearing FES instruments for prolonged periods. Additionally, prolonged FES usage may lead to muscle fatigue and decrease responsiveness, potentially affecting its effectiveness in stroke rehabilitation. Muscle fatigue presents a significant challenge associated with FES, imposing limitations on therapy duration and compromising its potential benefits (235). Furthermore, the effectiveness of conventional waveform modalities varies. These conventional waveforms lack guidance from neural movement control mechanisms, potentially reducing their efficacy in restoring movement (236, 237). Despite these concerns, strategies can be implemented to mitigate these drawbacks. For instance, proper electrode placement and adjustment of stimulation parameters can help optimize comfort and minimize muscle fatigue. Moreover, incorporating regular breaks during FES sessions and integrating FES with other rehabilitation modalities can help prevent overuse-related issues and enhance overall treatment outcomes. Recently, there has been interest in utilizing muscle synergy-driven FES waveforms, which have demonstrated effectiveness in restoring muscle function among stroke patients (238).

Furthermore, FES can be integrated into functional tasks or exercises, enabling stroke survivors to actively engage in movements with the assistance of electrical stimulation. This integration of FES with rehabilitation exercises promotes motor learning, supports task-specific training, and enhances functional recovery (234). The use of FES in stroke rehabilitation is well-established and considered a safe and effective adjunctive therapy. It complements other rehabilitation strategies, contributing to comprehensive treatment plans aimed at improving motor function and restoring independence in daily activities (239). Continued advancements in FES technology and its integration with other rehabilitation approaches hold significant promise for optimizing motor recovery outcomes, enhancing functional abilities, and improving the quality of life for individuals affected by stroke.

#### 7.3.4 Implantable devices (brain-computer interfaces)

Brain-computer interfaces (BCIs) represent cutting-edge implantable devices used in stroke rehabilitation to establish a direct communication link between the brain and external devices, aiming to restore motor function and enhance functional independence (240). In the context of stroke recovery, BCIs decode neural signals from the brain, allowing individuals to control external devices or prosthetics directly through their thoughts (241). This technology bypasses damaged neural pathways, enabling stroke survivors with severe motor impairments to interact with their environment or perform tasks that were previously compromised due to stroke-induced limitations (242). BCIs typically involve surgically implanting electrodes or sensors directly into the brain’s motor cortex or on the surface of the brain. These electrodes detect neural signals related to motor intentions or commands (243, 244). Through advanced algorithms and signal processing techniques, these neural signals are decoded into commands that can control external devices, such as robotic arms, computer interfaces, or assistive technology (245). The primary objective of BCIs in stroke rehabilitation is to restore motor function and improve independence in individuals with severe motor impairments (246). By interpreting neural signals and translating them into control commands for external devices, BCIs enable individuals to execute movements or interact with their environment based on their intention, bypassing the physical limitations caused by stroke-induced motor deficits (245). BCIs hold immense potential in stroke recovery by providing individuals with a means to regain control over their movements and perform tasks that were previously challenging or impossible (245). This technology aims to enhance functional abilities, promote independence in daily activities, and improve the overall quality of life for stroke survivors with severe motor impairments. Ongoing developments in BCI technology, such as improving signal accuracy, enhancing decoding algorithms, and refining implantation techniques, offer promising prospects for further optimizing their application in stroke rehabilitation. As research progresses, BCIs hold substantial promise for revolutionizing rehabilitation practices and empowering stroke survivors to regain functional abilities and independence.

## 8 Challenges and opportunities in stroke rehabilitation

### 8.1 Lack of standardized timing for stroke rehabilitation initiation

One of the challenges in stroke rehabilitation is the absence of standardized guidelines for the timing of initiating rehabilitation programs. The timing of rehabilitation can significantly influence recovery outcomes, yet there is considerable variability in clinical practice regarding when to start specific rehabilitation interventions.

The initiation of rehabilitation can vary based on several factors, including the type of stroke, the severity of the stroke, patient comorbidities, and the specific rehabilitation protocols being used. Some rehabilitation programs advocate for early initiation, typically within the first few days after a stroke, while others may recommend waiting until the patient is more medically stable. Early rehabilitation, which often begins within the first 24 to 48 h after stroke onset, aims to capitalize on the brain’s heightened plasticity during the acute phase. This approach can help prevent complications such as muscle atrophy and joint contractures and can improve functional outcomes (101). However, it requires careful monitoring to avoid overexertion and other risks. In contrast, some protocols suggest delaying the start of intensive rehabilitation until the subacute phase, typically 1 to 2 weeks post-stroke. This delay allows for better medical stabilization and assessment of the patient’s overall condition (247). Furthermore, the lack of standardized timing affects various rehabilitation programs differently. Physical therapy and early mobilization are often encouraged, but the intensity and type of exercises may vary based on the patient’s initial response and stability (123). The timing for starting occupational therapy can depend on the patient’s ability to participate in ADLs and cognitive readiness (248). Moreover, the initiation of speech and language therapy may be contingent on the patient’s neurological status and ability to engage in communication exercises (101). The variability in the initiation of rehabilitation programs underscores the need for standardized guidelines that can provide a framework for clinicians. Developing such guidelines would involve comprehensive research to determine the optimal timing for different rehabilitation interventions. Additionally, these guidelines should consider individual patient factors, such as age, comorbidities, and stroke severity. Collaboration among healthcare providers is also essential to establish a consensus on best practices. Thus, Addressing the lack of standardized timing for stroke rehabilitation initiation is crucial for improving patient outcomes. Establishing evidence-based guidelines can help ensure that patients receive timely and effective rehabilitation, tailored to their specific needs and conditions. Future research should focus on identifying the most effective timelines for various rehabilitation interventions to create a standardized approach that can be widely adopted in clinical practice.

### 8.2 Access to rehabilitation services

Access to comprehensive rehabilitation services remains a substantial challenge in stroke care. Geographical disparities, especially in rural or remote areas, often limit access to specialized rehabilitation centers and skilled healthcare professionals (249). Moreover, socioeconomic factors and financial constraints can impede access to rehabilitation, particularly for individuals with limited insurance coverage or financial resources (249). Addressing these challenges requires innovative approaches, such as telemedicine and mobile health solutions, to extend rehabilitation services beyond traditional clinical settings (250). Tele-rehabilitation programs, leveraging technology for remote assessment and therapy delivery, have shown promise in improving access to rehabilitation for underserved populations (251). Initiatives promoting community-based rehabilitation and partnerships with local healthcare providers play a crucial role in bridging the access gap, and ensuring equitable delivery of stroke rehabilitation services (252).

### 8.3 Stroke-related infections during hospitalization

Stroke patients are particularly vulnerable to infections during their hospital stay due to a combination of factors such as immobility, impaired swallowing (dysphagia), weakened immune function, and the need for invasive procedures (253). These infections can significantly impact patient outcomes, prolong hospitalization, and increase healthcare costs. The most common infections encountered during hospitalization of stroke patients include pneumonia, urinary tract infections (UTIs), and bloodstream infections (254).

Pneumonia is one of the most frequent complications in stroke patients, particularly those with dysphagia, which is difficulty in swallowing. Aspiration pneumonia occurs when food, liquid, or saliva is inhaled into the lungs instead of being swallowed into the esophagus. This risk is heightened in patients who have had a stroke because the neurological impairment can affect the muscles involved in swallowing (255). Pneumonia in stroke patients can lead to severe respiratory issues and prolong recovery times (256). Preventative measures include early assessment and management of swallowing difficulties, the use of thickened liquids, and positioning strategies during feeding to reduce the risk of aspiration. Urinary tract infections (UTIs) are another common complication in stroke patients. Factors contributing to UTIs include the use of urinary catheters, which are often necessary due to incontinence or mobility issues post-stroke. Catheter-associated urinary tract infections (CAUTIs) are a significant concern because they can lead to more serious systemic infections, including sepsis (257). Preventing UTIs involves careful catheter care, minimizing catheter use, and removing catheters as soon as possible. Hydration, proper hygiene, and regular monitoring for signs of infection are also crucial. Bloodstream infections, including central line-associated bloodstream infections (CLABSIs), can occur in stroke patients, especially those who require intravenous therapy or other invasive procedures. These infections can escalate into sepsis, a life-threatening response to infection, further complicating the patient’s recovery (258). To prevent bloodstream infections, healthcare providers must adhere to strict aseptic techniques during the insertion and maintenance of central lines and other invasive devices. Regular monitoring and prompt treatment of any signs of infection are essential.

Preventing infections in stroke patients requires a comprehensive and proactive approach. Encouraging early movement and physical therapy to reduce the risks associated with prolonged immobility, such as pneumonia and pressure ulcers. Mobilization helps improve circulation and lung function, reducing the likelihood of infections (256). Performing timely evaluations of swallowing ability to identify patients at risk for aspiration pneumonia. Interventions might include dietary modifications, swallowing exercises, and the use of thickened liquids (255). Implementing protocols to ensure the proper use and maintenance of urinary catheters. This includes adhering to guidelines for catheter insertion, ensuring cleanliness, and removing catheters as soon as they are no longer necessary to reduce the risk of CAUTIs (257). Promoting stringent hand hygiene practices among healthcare providers and visitors to minimize the transmission of pathogens in the hospital environment (258). Using antibiotics judiciously to prevent the development of antibiotic-resistant bacteria and reduce the risk of adverse reactions. This involves selecting the appropriate antibiotic and duration of treatment based on the specific infection and patient condition (257). Infections are a significant risk for stroke patients during hospitalization, contributing to higher morbidity and mortality rates. By implementing targeted preventive measures and closely monitoring patients, healthcare providers can reduce the incidence of infections and improve overall outcomes. Effective infection control practices are essential to ensure the best possible recovery for stroke patients.

### 8.4 Tailoring rehabilitation to individual needs

The heterogeneity of stroke-related impairments and the diverse needs of stroke survivors pose challenges in tailoring rehabilitation interventions. Standardized approaches may not adequately address the unique complexities of everyone’s recovery journey (47). Personalized rehabilitation plans that consider specific impairments, functional goals, cognitive abilities, and psychosocial factors are essential. Integrating advanced technologies, such as artificial intelligence (AI) and machine learning, into rehabilitation protocols enables the customization of interventions based on real-time patient data and responses (199, 259). Wearable devices and sensor-based technologies provide objective measures, facilitating the monitoring of progress and the adaptation of rehabilitation strategies according to individual needs (260). Establishing a framework for personalized rehabilitation that combines clinical expertise with technological advancements holds significant potential in optimizing outcomes for stroke survivors.

### 8.5 Interdisciplinary collaboration and training

Effective stroke rehabilitation requires seamless collaboration among diverse healthcare professionals, each contributing specialized expertise. However, achieving interdisciplinary collaboration poses challenges in terms of communication, coordination, and shared decision-making among multidisciplinary teams (261). Enhancing collaboration necessitates specialized training programs that emphasize interdisciplinary skills, fostering a shared understanding of stroke rehabilitation across disciplines. Training initiatives that promote team-based care, communication strategies, and shared decision-making frameworks are pivotal in overcoming barriers to collaboration (262). Moreover, establishing interprofessional education programs during healthcare training cultivates a collaborative mindset among future healthcare providers. Encouraging interdisciplinary rounds, case conferences, and joint care planning sessions facilitate cohesive care delivery, optimizing rehabilitation outcomes for stroke survivors (262).

In navigating these challenges, there exist promising opportunities to revolutionize stroke rehabilitation. By addressing access disparities, individualizing care, and fostering interdisciplinary collaboration, the stroke rehabilitation landscape can evolve to provide more comprehensive, patient-centered, and effective care for stroke survivors. Continued innovation, research, and collaboration across healthcare sectors hold the potential to significantly enhance stroke rehabilitation outcomes in the future.

## 9 Future directions in stroke rehabilitation

### 9.1 Personalized rehabilitation approaches

The future of stroke rehabilitation lies in advancing personalized approaches tailored to the specific needs and characteristics of each individual. Embracing innovative technologies, such as artificial intelligence and machine learning algorithms, holds immense promise in developing more sophisticated and adaptive rehabilitation protocols (19). These technologies can analyze vast datasets, including genomic, neuroimaging, and behavioral information, to create individualized treatment plans. Moreover, exploring the integration of virtual reality, augmented reality, and gamification strategies can further enhance engagement and motivation in personalized rehabilitation programs (263). The future direction emphasizes a shift towards precision medicine in stroke rehabilitation, catering treatments uniquely to each patient’s biological, cognitive, and psychosocial profile.

### 9.2 Integrating neuroplasticity concepts

Understanding and leveraging neuroplasticity principles are at the forefront of future stroke rehabilitation strategies. Emphasizing interventions that harness the brain’s inherent ability to reorganize and adapt following injury is crucial. Innovative therapeutic approaches, such as non-invasive brain stimulation techniques (e.g., transcranial magnetic stimulation, transcranial direct current stimulation), target neuroplastic changes in the brain (264). Additionally, combining traditional rehabilitation with novel strategies, such as enriched environments, sensory stimulation, and task-specific training, promotes neuroplasticity and enhances functional recovery. The future direction focuses on optimizing interventions that exploit neuroplasticity mechanisms to facilitate greater and more sustained recovery in stroke survivors.

### 9.3 Advancements in assistive technologies

The future landscape of stroke rehabilitation is intertwined with rapid advancements in assistive technologies aimed at augmenting functional abilities and improving independence. Cutting-edge innovations in robotics, exoskeletons, and wearable devices offer promising avenues to enhance motor recovery and support activities of daily living. Advancements in BCIs aim to seamlessly integrate with the brain’s neural signals, allowing for more intuitive control of assistive devices (265). Moreover, developments in sensor technology and smart home systems enable a more connected and supportive environment for stroke survivors. The future direction underscores the integration of these assistive technologies into rehabilitation programs, aiming to empower stroke survivors, enhance their autonomy, and improve overall quality of life. These future directions herald a paradigm shift in stroke rehabilitation, focusing on individualization, neuroplasticity-driven approaches, and the integration of innovative assistive technologies. Continued research, collaboration, and technological advancements hold substantial promise in revolutionizing stroke rehabilitation practices, ultimately improving outcomes and quality of life for individuals affected by stroke.

## 10 Conclusion

In conclusion, stroke rehabilitation represents a dynamic and evolving field that demands multidimensional approaches to address the diverse needs of stroke survivors. Throughout this manuscript, we have traversed various aspects of stroke rehabilitation, acknowledging the critical role of early identification, personalized interventions, and interdisciplinary collaboration in optimizing outcomes. While challenges persist, including access barriers and the complexity of tailoring interventions, the future of stroke rehabilitation appears promising. Future directions emphasizing personalized approaches, leveraging neuroplasticity concepts, and integrating cutting-edge assistive technologies offer new horizons in stroke care. By harnessing these opportunities and continuing to innovate, the rehabilitation community can significantly enhance recovery outcomes, foster greater independence, and ultimately improve the lives of those affected by stroke. This manuscript serves as a roadmap for future research, policy development, and clinical practice, underscoring the imperative of advancing stroke rehabilitation to meet the evolving needs of stroke survivors globally.

## Author contributions

XL: Data curation, Investigation, Methodology, Resources, Software, Validation, Writing – original draft. YH: Data curation, Investigation, Methodology, Project administration, Resources, Software, Validation, Writing – original draft. DW: Data curation, Supervision, Formal analysis, Investigation, Methodology, Project administration, Resources, Software, Writing – original draft. MR: Conceptualization, Investigation, Methodology, Project administration, Resources, Supervision, Visualization, Writing – original draft, Writing – review & editing.

## References

[1] Lindsay MP Norrving B Sacco RL Brainin M Hacke W Martins S. World Stroke Organization (WSO): global stroke fact sheet 2019. Int J Stroke. (2019) 14:806–17. doi: 10.1177/1747493019881353 31658892

[2] Feigin VL Stark BA Johnson CO Roth GA Bisignano C Abady GG. Global, regional, and national burden of stroke and its risk factors, 1990–2019: a systematic analysis for the Global Burden of Disease Study 2019. Lancet Neurol. (2021) 20:795–820. doi: 10.1016/S1474-4422(21)00252-0, PMID: 34487721 PMC8443449

[3] Owolabi MO Thrift AG Mahal A Ishida M Martins S Johnson WD. Primary stroke prevention worldwide: translating evidence into action. Lancet Public Health. (2022) 7:e74–85. doi: 10.1016/S2468-2667(21)00230-9, PMID: 34756176 PMC8727355

[4] Feigin VL Brainin M Norrving B Martins S Sacco RL Hacke W. World Stroke Organization (WSO): global stroke fact sheet 2022. Int J Stroke. (2022) 17:18–29. doi: 10.1177/17474930211065917, PMID: 34986727

[5] González-Santos J Rodríguez-Fernández P Pardo-Hernández R González-Bernal JJ Fernández-Solana J Santamaría-Peláez M. A cross-sectional study: determining factors of functional independence and quality of life of patients one month after having suffered a stroke. Int J Environ Res Public Health. (2023) 20:995. doi: 10.3390/ijerph20020995, PMID: 36673749 PMC9859177

[6] Freytes IM Sullivan M Schmitzberger M LeLaurin J Orozco T Eliazar-Macke N. Types of stroke-related deficits and their impact on family caregiver’s depressive symptoms, burden, and quality of life. Disabil Health J. (2021) 14:101019. doi: 10.1016/j.dhjo.2020.101019, PMID: 33187877

[7] Qureshi A Swain N Aldabe D Hale L. Exploring challenges affecting resilience in carers of stroke survivors: a qualitative descriptive study. Disabil Rehabil. (2023) 45:3696–704. doi: 10.1080/09638288.2022.2135774, PMID: 36269117

[8] Li S. Stroke recovery is a journey: prediction and potentials of motor recovery after a stroke from a practical perspective. Life. (2023) 13:2061. doi: 10.3390/life13102061, PMID: 37895442 PMC10608684

[9] Strilciuc S Grad DA Radu C Chira D Stan A Ungureanu M. The economic burden of stroke: a systematic review of cost of illness studies. J Med Life. (2021) 14:606–19. doi: 10.25122/jml-2021-0361, PMID: 35027963 PMC8742896

[10] Sun YA Phan H Buscot M-J Thrift AG Gall S. Area-level and individual-level socio-economic differences in health-related quality of life trajectories: results from a 10-year longitudinal stroke study. J Stroke Cerebrovasc Dis. (2023) 32:107188. doi: 10.1016/j.jstrokecerebrovasdis.2023.107188, PMID: 37216749

[11] Pandian JD Kalkonde Y Sebastian IA Felix C Urimubenshi G Bosch J. Stroke systems of care in low-income and middle-income countries: challenges and opportunities. Lancet. (2020) 396:1443–51. doi: 10.1016/S0140-6736(20)31374-X, PMID: 33129395

[12] Chimatiro GL Rhoda AJ. Scoping review of acute stroke care management and rehabilitation in low and middle-income countries. BMC Health Serv Res. (2019) 19:789. doi: 10.1186/s12913-019-4654-4 31684935 PMC6829977

[13] Owolabi MO Thrift AG Martins S Johnson W Pandian J Abd-Allah F. The state of stroke services across the globe: report of World Stroke Organization-World Health Organization surveys. Int J Stroke. (2021) 16:889–901. doi: 10.1177/17474930211019568, PMID: 33988062 PMC8800855

[14] Winstein CJ Stein J Arena R Bates B Cherney LR Cramer SC. Guidelines for adult stroke rehabilitation and recovery: a guideline for healthcare professionals from the American Heart Association/American Stroke Association. Stroke. (2016) 47:e98–e169. doi: 10.1161/STR.0000000000000098, PMID: 27145936

[15] Goldstein LB Bushnell CD Adams RJ Appel LJ Braun LT Chaturvedi S. Guidelines for the primary prevention of stroke: a guideline for healthcare professionals from the American Heart Association/American Stroke Association. Stroke. (2011) 42:517–84. doi: 10.1161/STR.0b013e3181fcb238 21127304

[16] Young JA Tolentino M. Stroke evaluation and treatment. Top Stroke Rehabil. (2009) 16:389–410. doi: 10.1310/tsr1606-389 20139041

[17] Meyer BC Hemmen TM Jackson CM Lyden PD. Modified National Institutes of Health Stroke Scale for use in stroke clinical trials: prospective reliability and validity. Stroke. (2002) 33:1261–6. doi: 10.1161/01.STR.0000015625.87603.A7 11988601

[18] Ringleb P Schellinger P Schranz C Hacke W. Thrombolytic therapy within 3 to 6 hours after onset of ischemic stroke: useful or harmful? Stroke. (2002) 33:1437–41. doi: 10.1161/01.STR.0000015555.21285.DB 11988629

[19] Saceleanu VM Toader C Ples H Covache-Busuioc R-A Costin HP Bratu B-G. Integrative approaches in acute ischemic stroke: from symptom recognition to future innovations. Biomedicines. (2023) 11:2617. doi: 10.3390/biomedicines11102617, PMID: 37892991 PMC10604797

[20] Di Carli MF Geva T Davidoff R. The future of cardiovascular imaging. Circulation. (2016) 133:2640–61. doi: 10.1161/CIRCULATIONAHA.116.023511 27324360

[21] Vilela P Rowley HA. Brain ischemia: CT and MRI techniques in acute ischemic stroke. Eur J Radiol. (2017) 96:162–72. doi: 10.1016/j.ejrad.2017.08.014 29054448

[22] Wang K Shou Q Ma SJ Liebeskind D Qiao XJ Saver J. Deep learning detection of penumbral tissue on arterial spin labeling in stroke. Stroke. (2020) 51:489–97. doi: 10.1161/STROKEAHA.119.027457, PMID: 31884904 PMC7224203

[23] Tarpley J Franc D Tansy AP Liebeskind DS. Use of perfusion imaging and other imaging techniques to assess risks/benefits of acute stroke interventions. Curr Atheroscler Rep. (2013) 15:336. doi: 10.1007/s11883-013-0336-6 23666875 PMC3683532

[24] Catanese L Tarsia J Fisher M. Acute ischemic stroke therapy overview. Circ Res. (2017) 120:541–58. doi: 10.1161/CIRCRESAHA.116.309278 28154103

[25] Abou-Chebl A. Management of acute ischemic stroke. Curr Cardiol Rep. (2013) 15:348. doi: 10.1007/s11886-013-0348-4 23420444

[26] Gralla J Brekenfeld C Mordasini P Schroth G. Mechanical thrombolysis and stenting in acute ischemic stroke. Stroke. (2012) 43:280–5. doi: 10.1161/STROKEAHA.111.626903 22198985

[27] Campbell BC De Silva DA Macleod MR Coutts SB Schwamm LH Davis SM. Ischaemic stroke. Nat Rev Dis Primers. (2019) 5:70. doi: 10.1038/s41572-019-0118-8 31601801

[28] Struffert T Deuerling-Zheng Y Kloska S Engelhorn T Strother C Kalender W. Flat detector CT in the evaluation of brain parenchyma, intracranial vasculature, and cerebral blood volume: a pilot study in patients with acute symptoms of cerebral ischemia. Am J Neuroradiol. (2010) 31:1462–9. doi: 10.3174/ajnr.A2083, PMID: 20378700 PMC7966101

[29] Roach ES Golomb MR Adams R Biller J Daniels S Deveber G. Management of stroke in infants and children: a scientific statement from a Special Writing Group of the American Heart Association Stroke Council and the Council on Cardiovascular Disease in the Young. Stroke. (2008) 39:2644–91. doi: 10.1161/STROKEAHA.108.189696 18635845

[30] Qazi E Al-Ajlan FS Najm M Menon BK. The role of vascular imaging in the initial assessment of patients with acute ischemic stroke. Curr Neurol Neurosci Rep. (2016) 16:32. doi: 10.1007/s11910-016-0632-y 26898684

[31] Willie C Colino F Bailey D Tzeng Y Binsted G Jones L. Utility of transcranial Doppler ultrasound for the integrative assessment of cerebrovascular function. J Neurosci Methods. (2011) 196:221–37. doi: 10.1016/j.jneumeth.2011.01.011, PMID: 21276818

[32] Wechsler LR Demaerschalk BM Schwamm LH Adeoye OM Audebert HJ Fanale CV. Telemedicine quality and outcomes in stroke: a scientific statement for healthcare professionals from the American Heart Association/American Stroke Association. Stroke. (2017) 48:e3–e25. doi: 10.1161/STR.0000000000000114 27811332

[33] Yedavalli VS Tong E Martin D Yeom KW Forkert ND. Artificial intelligence in stroke imaging: current and future perspectives. Clin Imaging. (2021) 69:246–54. doi: 10.1016/j.clinimag.2020.09.005, PMID: 32980785

[34] Lukić S Ćojbasić Ž Perić Z Milošević Z Spasić M Pavlović V. Artificial neural networks based early clinical prediction of mortality after spontaneous intracerebral hemorrhage. Acta Neurol Belg. (2012) 112:375–82. doi: 10.1007/s13760-012-0093-2, PMID: 22674031

[35] Wang H-L Hsu W-Y Lee M-H Weng H-H Chang S-W Yang J-T. Automatic machine-learning-based outcome prediction in patients with primary intracerebral hemorrhage. Front Neurol. (2019) 10:910. doi: 10.3389/fneur.2019.00910, PMID: 31496988 PMC6713018

[36] Xu X Zhang J Yang K Wang Q Chen X Xu B. Prognostic prediction of hypertensive intracerebral hemorrhage using CT radiomics and machine learning. Brain Behav. (2021) 11:e02085. doi: 10.1002/brb3.2085, PMID: 33624945 PMC8119849

[37] Chen Y Jiang C Chang J Qin C Zhang Q Ye Z. An artificial intelligence-based prognostic prediction model for hemorrhagic stroke. Eur J Radiol. (2023) 167:111081. doi: 10.1016/j.ejrad.2023.111081, PMID: 37716178

[38] Adams HP Jr Lyden P. Assessment of a patient with stroke: neurological examination and clinical rating scales. Handb Clin Neurol. (2008) 94:971–1009. doi: 10.1016/S0072-9752(08)94048-3 18793885

[39] Henderson D Jensen M Drucker J Lutz A. Rehabilitation of speech, language, and swallowing disorders in clients with acquired brain injury In: Elbaum J, editor. Acquired brain injury. Cham: Springer (2019). 201–26.

[40] Jauch EC Saver JL Adams HP Jr Bruno A Connors J Demaerschalk BM. Guidelines for the early management of patients with acute ischemic stroke: a guideline for healthcare professionals from the American Heart Association/American Stroke Association. Stroke. (2013) 44:870–947. doi: 10.1161/STR.0b013e318284056a 23370205

[41] Mutai H Furukawa T Araki K Misawa K Hanihara T. Factors associated with functional recovery and home discharge in stroke patients admitted to a convalescent rehabilitation ward. Geriatr Gerontol Int. (2012) 12:215–22. doi: 10.1111/j.1447-0594.2011.00747.x, PMID: 21929733

[42] Culebras A Kase CS Masdeu JC Fox AJ Bryan RN Grossman CB. Practice guidelines for the use of imaging in transient ischemic attacks and acute stroke. A report of the Stroke Council, American Heart Association. Stroke. (1997) 28:1480–97. doi: 10.1161/01.STR.28.7.1480, PMID: 9227705

[43] Kim Y-H. Rehabilitation after hemorrhagic stroke: from acute to chronic stage In: Lee SH, editor. Stroke revisited: hemorrhagic stroke. Singapore: Springer (2018). 219–31.

[44] Coleman ER Moudgal R Lang K Hyacinth HI Awosika OO Kissela BM. Early rehabilitation after stroke: a narrative review. Curr Atheroscler Rep. (2017) 19:59. doi: 10.1007/s11883-017-0686-6 29116473 PMC5802378

[45] Dobkin BH. Strategies for stroke rehabilitation. Lancet Neurol. (2004) 3:528–36. doi: 10.1016/S1474-4422(04)00851-8, PMID: 15324721 PMC4164204

[46] Chen L Xiao LD De Bellis A. First-time stroke survivors and caregivers’ perceptions of being engaged in rehabilitation. J Adv Nurs. (2016) 72:73–84. doi: 10.1111/jan.12819, PMID: 26399942

[47] Miller EL Murray L Richards L Zorowitz RD Bakas T Clark P. Comprehensive overview of nursing and interdisciplinary rehabilitation care of the stroke patient: a scientific statement from the American Heart Association. Stroke. (2010) 41:2402–48. doi: 10.1161/STR.0b013e3181e7512b, PMID: 20813995

[48] Han J Anson J Waddington G Adams R. Proprioceptive performance of bilateral upper and lower limb joints: side-general and site-specific effects. Exp Brain Res. (2013) 226:313–23. doi: 10.1007/s00221-013-3437-0, PMID: 23423167 PMC3627017

[49] Oguz S Demirbuken I Kavlak B Acar G Yurdalan SU Polat MG. The relationship between objective balance, perceived sense of balance, and fear of falling in stroke patients. Top Stroke Rehabil. (2017) 24:527–32. doi: 10.1080/10749357.2017.1322251, PMID: 28472895

[50] Cayco CS Gorgon EJR Lazaro RT. Effects of proprioceptive neuromuscular facilitation on balance, strength, and mobility of an older adult with chronic stroke: a case report. J Bodyw Mov Ther. (2017) 21:767–74. doi: 10.1016/j.jbmt.2016.10.008, PMID: 29037625

[51] Plummer P Eskes G Wallace S Giuffrida C Fraas M Campbell G. Cognitive-motor interference during functional mobility after stroke: state of the science and implications for future research. Arch Phys Med Rehabil. (2013) 94:2565–2574.e6. doi: 10.1016/j.apmr.2013.08.002, PMID: 23973751 PMC3842379

[52] Silsupadol P Lugade V Shumway-Cook A van Donkelaar P Chou L-S Mayr U. Training-related changes in dual-task walking performance of elderly persons with balance impairment: a double-blind, randomized controlled trial. Gait Posture. (2009) 29:634–9. doi: 10.1016/j.gaitpost.2009.01.006, PMID: 19201610 PMC2707497

[53] Tasseel-Ponche S Roussel M Toba MN Sader T Barbier V Delafontaine A. Dual-task versus single-task gait rehabilitation after stroke: the protocol of the cognitive-motor synergy multicenter, randomized, controlled superiority trial (SYNCOMOT). Trials. (2023) 24:172. doi: 10.1186/s13063-023-07138-x 36890548 PMC9994785

[54] Yang Z-Q Wei M-F Chen L Xi J-N. Research progress in the application of motor-cognitive dual-task training in rehabilitation of walking function in stroke patients. J Neurorestoratol. (2023) 11:100028. doi: 10.1016/j.jnrt.2022.100028

[55] Langhorne P Coupar F Pollock A. Motor recovery after stroke: a systematic review. Lancet Neurol. (2009) 8:741–54. doi: 10.1016/S1474-4422(09)70150-4 19608100

[56] Aderinto N Olatunji G Abdulbasit MO Edun M Aboderin G Egbunu E. Exploring the efficacy of virtual reality-based rehabilitation in stroke: a narrative review of current evidence. Ann Med. (2023) 55:2285907. doi: 10.1080/07853890.2023.2285907, PMID: 38010358 PMC10836287

[57] Verschure PF Dos Santos FP Sharma V. Redefining stroke rehabilitation: mobilizing the embodied goal-oriented brain. Curr Opin Neurobiol. (2023) 83:102807. doi: 10.1016/j.conb.2023.102807 37980804

[58] Moon J-H Park K-Y Kim H-J Na C-H. The effects of task-oriented circuit training using rehabilitation tools on the upper-extremity functions and daily activities of patients with acute stroke: a randomized controlled pilot trial. Osong Public Health Res Perspect. (2018) 9:225–30. doi: 10.24171/j.phrp.2018.9.5.03, PMID: 30402377 PMC6202022

[59] Wolf SL Winstein CJ Miller JP Taub E Uswatte G Morris D. Effect of constraint-induced movement therapy on upper extremity function 3 to 9 months after stroke the EXCITE randomized clinical trial. JAMA. (2006) 296:2095–104. doi: 10.1001/jama.296.17.2095, PMID: 17077374

[60] Mushtaq W Hamdani N Noohu MM Raghavan S. Effect of modified constrain induced movement therapy on fatigue and motor performance in sub acute stroke. J Stroke Cerebrovasc Dis. (2020) 29:105378. doi: 10.1016/j.jstrokecerebrovasdis.2020.105378 33080562

[61] Shi YX Tian JH Yang KH Zhao Y. Modified constraint-induced movement therapy versus traditional rehabilitation in patients with upper-extremity dysfunction after stroke: a systematic review and meta-analysis. Arch Phys Med Rehabil. (2011) 92:972–82. doi: 10.1016/j.apmr.2010.12.036 21621674

[62] Barzel A Ketels G Stark A Tetzlaff B Daubmann A Wegscheider K. Home-based constraint-induced movement therapy for patients with upper limb dysfunction after stroke (HOMECIMT): a cluster-randomised, controlled trial. Lancet Neurol. (2015) 14:893–902. doi: 10.1016/S1474-4422(15)00147-7 26231624

[63] Wu J Cheng H Zhang J Yang S Cai S. Robot-assisted therapy for upper extremity motor impairment after stroke: a systematic review and meta-analysis. Phys Ther. (2021) 101:pzab010. doi: 10.1093/ptj/pzab010, PMID: 33454787

[64] Jiang S You H Zhao W Zhang M. Effects of short-term upper limb robot-assisted therapy on the rehabilitation of sub-acute stroke patients. Technol Health Care. (2021) 29:295–303. doi: 10.3233/THC-202127, PMID: 33285652

[65] Cho K-H Song W-K. Effects of two different robot-assisted arm training on upper limb motor function and kinematics in chronic stroke survivors: a randomized controlled trial. Top Stroke Rehabil. (2021) 28:241–50. doi: 10.1080/10749357.2020.1804699, PMID: 32791945

[66] Lo AC Guarino PD Richards LG Haselkorn JK Wittenberg GF Federman DG. Robot-assisted therapy for long-term upper-limb impairment after stroke. N Engl J Med. (2010) 362:1772–83. doi: 10.1056/NEJMoa0911341, PMID: 20400552 PMC5592692

[67] Peng Q-c Yin L Cao Y. Effectiveness of virtual reality in the rehabilitation of motor function of patients with subacute stroke: a meta-analysis. Front Neurol. (2021) 12:639535. doi: 10.3389/fneur.2021.639535, PMID: 34025553 PMC8131676

[68] Subramanian SK Lourenço CB Chilingaryan G Sveistrup H Levin MF. Arm motor recovery using a virtual reality intervention in chronic stroke: randomized control trial. Neurorehabil Neural Repair. (2013) 27:13–23. doi: 10.1177/1545968312449695 22785001

[69] Laver K. Virtual reality for stroke rehabilitation In: Virtual reality in health and rehabilitation. Boca Raton: CRC Press (2020). 19–28.

[70] Billinger SA Arena R Bernhardt J Eng JJ Franklin BA Johnson CM. Physical activity and exercise recommendations for stroke survivors: a statement for healthcare professionals from the American Heart Association/American Stroke Association. Stroke. (2014) 45:2532–53. doi: 10.1161/STR.0000000000000022 24846875

[71] Marzolini S Robertson AD Oh P Goodman JM Corbett D Du X. Aerobic training and mobilization early post-stroke: cautions and considerations. Front Neurol. (2019) 10:1187. doi: 10.3389/fneur.2019.01187, PMID: 31803129 PMC6872678

[72] Pang MY Charlesworth SA Lau RW Chung RC. Using aerobic exercise to improve health outcomes and quality of life in stroke: evidence-based exercise prescription recommendations. Cerebrovasc Dis. (2013) 35:7–22. doi: 10.1159/000346075, PMID: 23428993

[73] Steultjens EM Dekker J Bouter LM van de Nes JC Cup EH van den Ende CH. Occupational therapy for stroke patients: a systematic review. Stroke. (2003) 34:676–87. doi: 10.1161/01.STR.0000057576.77308.30 12624291

[74] De-Rosende-Celeiro I Rey-Villamayor A Francisco-de-Miguel I Ávila-Álvarez A. Independence in daily activities after stroke among occupational therapy patients and its relationship with unilateral neglect. Int J Environ Res Public Health. (2021) 18:7537. doi: 10.3390/ijerph18147537, PMID: 34299988 PMC8306679

[75] Fauth EB Schaefer SY Zarit SH Ernsth-Bravell M Johansson B. Associations between fine motor performance in activities of daily living and cognitive ability in a nondemented sample of older adults: implications for geriatric physical rehabilitation. J Aging Health. (2017) 29:1144–59. doi: 10.1177/0898264316654674, PMID: 27339106 PMC5179315

[76] Bhogal SK Teasell R Speechley M. Intensity of aphasia therapy, impact on recovery. Stroke. (2003) 34:987–93. doi: 10.1161/01.STR.0000062343.64383.D0, PMID: 12649521

[77] Dilworth C. The role of the speech language pathologist in acute stroke. Ann Indian Acad Neurol. (2008) 11:108–S18. doi: 10.4103/0972-2327.41724 PMC9204112 35721441

[78] Lazar RM Boehme AK. Aphasia as a predictor of stroke outcome. Curr Neurol Neurosci Rep. (2017) 17:83. doi: 10.1007/s11910-017-0797-z 28929424 PMC13077792

[79] Eraifej J Clark W France B Desando S Moore D. Effectiveness of upper limb functional electrical stimulation after stroke for the improvement of activities of daily living and motor function: a systematic review and meta-analysis. Syst Rev. (2017) 6:40. doi: 10.1186/s13643-017-0435-5 28245858 PMC5331643

[80] Quandt F Hummel FC. The influence of functional electrical stimulation on hand motor recovery in stroke patients: a review. Exp Transl Stroke Med. (2014) 6:9. doi: 10.1186/2040-7378-6-9 25276333 PMC4178310

[81] Marquez-Chin C Popovic MR. Functional electrical stimulation therapy for restoration of motor function after spinal cord injury and stroke: a review. Biomed Eng Online. (2020) 19:34. doi: 10.1186/s12938-020-00773-4, PMID: 32448143 PMC7245767

[82] Vafadar AK Côté JN Archambault PS. Effectiveness of functional electrical stimulation in improving clinical outcomes in the upper arm following stroke: a systematic review and meta-analysis. Biomed Res Int. (2015) 2015:729768. doi: 10.1155/2015/729768 25685805 PMC4317587

[83] Thieme H Morkisch N Mehrholz J Pohl M Behrens J Borgetto B. Mirror therapy for improving motor function after stroke. Cochrane Database Syst Rev. (2018) 2018:CD008449. doi: 10.1002/14651858.CD008449.pub3 PMC6513639 29993119

[84] Yavuzer G Selles R Sezer N Sütbeyaz S Bussmann JB Köseoğlu F. Mirror therapy improves hand function in subacute stroke: a randomized controlled trial. Arch Phys Med Rehabil. (2008) 89:393–8. doi: 10.1016/j.apmr.2007.08.162, PMID: 18295613

[85] Gandhi DB Sterba A Khatter H Pandian JD. Mirror therapy in stroke rehabilitation: current perspectives. Ther Clin Risk Manag. (2020) 16:75–85. doi: 10.2147/TCRM.S206883, PMID: 32103968 PMC7012218

[86] Hartman K Altschuler EL. Mirror therapy for hemiparesis following stroke: a review. Curr Phys Med Rehabil Rep. (2016) 4:237–48. doi: 10.1007/s40141-016-0131-8

[87] Cumming TB Marshall RS Lazar RM. Stroke, cognitive deficits, and rehabilitation: still an incomplete picture. Int J Stroke. (2013) 8:38–45. doi: 10.1111/j.1747-4949.2012.00972.x, PMID: 23280268

[88] Cicerone KD Goldin Y Ganci K Rosenbaum A Wethe JV Langenbahn DM. Evidence-based cognitive rehabilitation: systematic review of the literature from 2009 through 2014. Arch Phys Med Rehabil. (2019) 100:1515–33. doi: 10.1016/j.apmr.2019.02.011, PMID: 30926291

[89] Cicerone KD Langenbahn DM Braden C Malec JF Kalmar K Fraas M. Evidence-based cognitive rehabilitation: updated review of the literature from 2003 through 2008. Arch Phys Med Rehabil. (2011) 92:519–30. doi: 10.1016/j.apmr.2010.11.015, PMID: 21440699

[90] Draaisma LR Wessel MJ Hummel FC. Non-invasive brain stimulation to enhance cognitive rehabilitation after stroke. Neurosci Lett. (2020) 719:133678. doi: 10.1016/j.neulet.2018.06.047, PMID: 29960054

[91] Marinho-Buzelli AR Bonnyman AM Verrier MC. The effects of aquatic therapy on mobility of individuals with neurological diseases: a systematic review. Clin Rehabil. (2015) 29:741–51. doi: 10.1177/0269215514556297, PMID: 25394397

[92] Giuriati S Servadio A Temperoni G Curcio A Valente D Galeoto G. The effect of aquatic physical therapy in patients with stroke: a systematic review and meta-analysis. Top Stroke Rehabil. (2021) 28:19–32. doi: 10.1080/10749357.2020.1755816 32340581

[93] Grau-Sánchez J Segura E Sanchez-Pinsach D Raghavan P Münte TF Palumbo AM. Enriched music-supported therapy for chronic stroke patients: a study protocol of a randomised controlled trial. BMC Neurol. (2021) 21:19. doi: 10.1186/s12883-020-02019-1 33435919 PMC7801568

[94] Grau-Sánchez J Münte TF Altenmüller E Duarte E Rodríguez-Fornells A. Potential benefits of music playing in stroke upper limb motor rehabilitation. Neurosci Biobehav Rev. (2020) 112:585–99. doi: 10.1016/j.neubiorev.2020.02.027, PMID: 32092314

[95] Altenmüller E James CE. The impact of music interventions on motor rehabilitation following stroke in elderly In: Cuddy LL Belleville S Moussard A, editors. Music and the aging brain. CA, USA: Elsevier Academic Press (2020). 407–32.

[96] van Nimwegen D Hjelle EG Bragstad LK Kirkevold M Sveen U Hafsteinsdóttir T. Interventions for improving psychosocial well-being after stroke: a systematic review. Int J Nurs Stud. (2023) 142:104492. doi: 10.1016/j.ijnurstu.2023.104492, PMID: 37084476

[97] Hackett ML Anderson CS House A Xia J. Interventions for treating depression after stroke. Cochrane Database Syst Rev. (2008) 2008:CD003437. doi: 10.1002/14651858.CD003437.pub3 18843644

[98] Kirkevold M Kildal Bragstad L Bronken BA Kvigne K Martinsen R Gabrielsen Hjelle E. Promoting psychosocial well-being following stroke: study protocol for a randomized, controlled trial. BMC Psychol. (2018) 6:1–12. doi: 10.1186/s40359-018-0223-6 29615136 PMC5883408

[99] Sommer CJ Schäbitz W-R. Principles and requirements for stroke recovery science. J Cereb Blood Flow Metab. (2021) 41:471–85. doi: 10.1177/0271678X20970048, PMID: 33175596 PMC7907998

[100] Hasan TF Hasan H Kelley RE. Overview of acute ischemic stroke evaluation and management. Biomedicines. (2021) 9:1486. doi: 10.3390/biomedicines9101486, PMID: 34680603 PMC8533104

[101] Langhorne P Bernhardt J Kwakkel G. Stroke rehabilitation. Lancet. (2011) 377:1693–702. doi: 10.1016/S0140-6736(11)60325-5 21571152

[102] Boyd LA Hayward KS Ward NS Stinear CM Rosso C Fisher RJ. Biomarkers of stroke recovery: consensus-based core recommendations from the stroke recovery and rehabilitation roundtable. Int J Stroke. (2017) 12:480–93. doi: 10.1177/1747493017714176, PMID: 28697711 PMC6791523

[103] Todhunter-Brown A Baer G Campbell P Choo PL Forster A Morris J. Physical rehabilitation approaches for the recovery of function and mobility following stroke. Cochrane Database Syst Rev. (2014) 2014:CD001920. doi: 10.1002/14651858.CD001920.pub3 24756870 PMC6465059

[104] Ekechukwu END Olowoyo P Nwankwo KO Olaleye OA Ogbodo VE Hamzat TK. Pragmatic solutions for stroke recovery and improved quality of life in low-and middle-income countries—a systematic review. Front Neurol. (2020) 11:531709. doi: 10.3389/fneur.2020.00337 PMC7336355 32695058

[105] Chiaramonte R Bonfiglio M Leonforte P Coltraro GL Guerrera CS Vecchio M. Proprioceptive and dual-task training: the key of stroke rehabilitation, a systematic review. J Funct Morphol Kinesiol. (2022) 7:53. doi: 10.3390/jfmk7030053, PMID: 35893327 PMC9326539

[106] Brady MC Godwin J Enderby P Kelly H Campbell P. Speech and language therapy for aphasia after stroke: an updated systematic review and meta-analyses. Stroke. (2016) 47:e236–7. doi: 10.1161/STROKEAHA.116.014439

[107] Mukherjee D Levin RL Heller W. The cognitive, emotional, and social sequelae of stroke: psychological and ethical concerns in post-stroke adaptation. Top Stroke Rehabil. (2006) 13:26–35. doi: 10.1310/tsr1304-26, PMID: 17082166

[108] Veerbeek JM Langbroek-Amersfoort AC Van Wegen EE Meskers CG Kwakkel G. Effects of robot-assisted therapy for the upper limb after stroke: a systematic review and meta-analysis. Neurorehabil Neural Repair. (2017) 31:107–21. doi: 10.1177/1545968316666957 27597165

[109] Thieme H Mehrholz J Pohl M Behrens J Dohle C. Mirror therapy for improving motor function after stroke. Stroke. (2013) 44:e1–2. doi: 10.1161/STROKEAHA.112.673087, PMID: 23390640

[110] Magee WL Clark I Tamplin J Bradt J. Music interventions for acquired brain injury. Cochrane Database Syst Rev. (2017) 2017:CD006787. doi: 10.1002/14651858.CD006787.pub3 PMC6464962 28103638

[111] Cumming TB Packer M Kramer SF English C. The prevalence of fatigue after stroke: a systematic review and meta-analysis. Int J Stroke. (2016) 11:968–77. doi: 10.1177/1747493016669861, PMID: 27703065

[112] Boulanger J Lindsay M Gubitz G Smith E Stotts G Foley N. Canadian stroke best practice recommendations for acute stroke management: prehospital, emergency department, and acute inpatient stroke care, update 2018. Int J Stroke. (2018) 13:949–84. doi: 10.1177/1747493018786616 30021503

[113] Hurford R Sekhar A Hughes TA Muir KW. Diagnosis and management of acute ischaemic stroke. Pract Neurol. (2020) 20:304–16. doi: 10.1136/practneurol-2020-002557, PMID: 32507747 PMC7577107

[114] Seidel PM Seidel GK. Stroke rehabilitation In: Hans S, editor. Extracranial carotid and vertebral artery disease. Cham: Springer (2018). 279–92.

[115] Rowland TJ Cooke DM Gustafsson LA. Role of occupational therapy after stroke. Ann Indian Acad Neurol. (2008) 11:S99–S107. PMID: 35721442 PMC9204113

[116] Armstrong JR Mosher BD. Aspiration pneumonia after stroke: intervention and prevention. Neurohospitalist. (2011) 1:85–93. doi: 10.1177/1941875210395775 23983842 PMC3726080

[117] Kalra L. Medical complications after stroke In: Stroke recovery and rehabilitation. CA, USA (2008). 405.

[118] Adams HP Jr Del Zoppo G Alberts MJ Bhatt DL Brass L Furlan A. Guidelines for the early management of adults with ischemic stroke: a guideline from the American Heart Association/American Stroke Association Stroke Council, Clinical Cardiology Council, Cardiovascular Radiology and Intervention Council, and the Atherosclerotic Peripheral Vascular Disease and Quality of Care Outcomes in Research Interdisciplinary Working Groups: the American Academy of Neurology affirms the value of this guideline as an educational tool for neurologists. Stroke. (2007) 38:1655–711. doi: 10.1161/STROKEAHA.107.181486, PMID: 17431204

[119] Norrving B Barrick J Davalos A Dichgans M Cordonnier C Guekht A. Action plan for stroke in Europe 2018–2030. Eur Stroke J. (2018) 3:309–36. doi: 10.1177/2396987318808719, PMID: 31236480 PMC6571507

[120] Beebe JA Lang CE. Active range of motion predicts upper extremity function 3 months after stroke. Stroke. (2009) 40:1772–9. doi: 10.1161/STROKEAHA.108.536763, PMID: 19265051 PMC2718540

[121] van der Brugge F. Influenceable functions and cognitive rehabilitation In: Neurorehabilitation for central nervous system disorders (2018). 91–117.

[122] Rhoades D Bergman C Pasquina PF. Rehabilitation in the setting of neurotrauma In: Ecklund J Moores L, editors. Neurotrauma management for the severely injured polytrauma patient. Cham: Springer (2017). 255–77.

[123] Bernhardt J English C Johnson L Cumming TB. Early mobilization after stroke: early adoption but limited evidence. Stroke. (2015) 46:1141–6. doi: 10.1161/STROKEAHA.114.007434 25690544

[124] Tinga AM Visser-Meily JMA van der Smagt MJ Van der Stigchel S van Ee R Nijboer TCW. Multisensory stimulation to improve low-and higher-level sensory deficits after stroke: a systematic review. Neuropsychol Rev. (2016) 26:73–91. doi: 10.1007/s11065-015-9301-1, PMID: 26490254 PMC4762927

[125] Barritt AW Smithard DG. Role of cerebral cortex plasticity in the recovery of swallowing function following dysphagic stroke. Dysphagia. (2009) 24:83–90. doi: 10.1007/s00455-008-9162-3, PMID: 18716838

[126] Bonini-Rocha AC de Andrade ALS Moraes AM Matheus LBG Diniz LR Martins WR. Effectiveness of circuit-based exercises on gait speed, balance, and functional mobility in people affected by stroke: a meta-analysis. PM R. (2018) 10:398–409. doi: 10.1016/j.pmrj.2017.09.014, PMID: 29111465

[127] Becker BE. Aquatic therapy: scientific foundations and clinical rehabilitation applications. PM R. (2009) 1:859–72. doi: 10.1016/j.pmrj.2009.05.017 19769921

[128] Aichner F Adelwöhrer C Haring H-P. Rehabilitation approaches to stroke In: Fleischhacker WW Brooks DJ, editors. Stroke-vascular diseases. Vienna: Springer (2002) 10.1007/978-3-7091-6137-1_4 12597609

[129] Fluet GG Roy D Llorens R. Basis and clinical evidence of virtual reality-based rehabilitation of sensorimotor impairments after stroke In: Reinkensmeyer DJ Marchal-Crespo L Dietz V, editors. Neurorehabilitation technology. Cham: Springer (2022). 429–66.

[130] Adeoye O Nyström KV Yavagal DR Luciano J Nogueira RG Zorowitz RD. Recommendations for the establishment of stroke systems of care: a 2019 update: a policy statement from the American Stroke Association. Stroke. (2019) 50:e187–210. doi: 10.1161/STR.0000000000000173 31104615

[131] Indar A Nelson M Berta W Mylopoulos M. A critical appraisal of professional competency frameworks: what guidance is provided for stroke rehabilitation clinicians managing ‘complexity’? J Multimorb Comorb. (2023) 13:26335565231215671. doi: 10.1177/26335565231215671, PMID: 38024541 PMC10657527

[132] Hersh D Worrall L Howe T Sherratt S Davidson B. SMARTER goal setting in aphasia rehabilitation. Aphasiology. (2012) 26:220–33. doi: 10.1080/02687038.2011.640392

[133] Calvaresi D Calbimonte J-P. Real-time compliant stream processing agents for physical rehabilitation. Sensors. (2020) 20:746. doi: 10.3390/s20030746, PMID: 32013222 PMC7038372

[134] Dobkin BH. A rehabilitation-internet-of-things in the home to augment motor skills and exercise training. Neurorehabil Neural Repair. (2017) 31:217–27. doi: 10.1177/1545968316680490 27885161 PMC5315644

[135] Johnston V Brakenridge C Valiant D Ling CLK Andrews N Gane EM. Using framework analysis to understand multiple stakeholders’ views of vocational rehabilitation following acquired brain injury. Brain Impair. (2023) 24:347–70. doi: 10.1017/BrImp.2022.27, PMID: 38167182

[136] Teasell RW Foley NC Bhogal SK Speechley MR. An evidence-based review of stroke rehabilitation. Top Stroke Rehabil. (2003) 10:29–58. doi: 10.1310/8YNA-1YHK-YMHB-XTE1 12970830

[137] Duncan PW Zorowitz R Bates B Choi JY Glasberg JJ Graham GD. Management of adult stroke rehabilitation care: a clinical practice guideline. Stroke. (2005) 36:e100–43. doi: 10.1161/01.STR.0000180861.54180.FF, PMID: 16120836

[138] Latham NK Jette DU Slavin M Richards LG Procino A Smout RJ. Physical therapy during stroke rehabilitation for people with different walking abilities. Arch Phys Med Rehabil. (2005) 86:41–50. doi: 10.1016/j.apmr.2005.08.128 16373139

[139] Sullivan JE Crowner BE Kluding PM Nichols D Rose DK Yoshida R. Outcome measures for individuals with stroke: process and recommendations from the American Physical Therapy Association neurology section task force. Phys Ther. (2013) 93:1383–96. doi: 10.2522/ptj.20120492, PMID: 23704035

[140] Charalambous CC Bonilha HS Kautz SA Gregory CM Bowden MG. Rehabilitating walking speed poststroke with treadmill-based interventions: a systematic review of randomized controlled trials. Neurorehabil Neural Repair. (2013) 27:709–21. doi: 10.1177/1545968313491005, PMID: 23764885 PMC4478607

[141] Wu M. A flexible cable-driven robotic system: design and its clinical application for improving walking function in adults with stroke, SCI, and children with CP In: Reinkensmeyer DJ Marchal-Crespo L Dietz V, editors. Neurorehabilitation technology. Cham: Springer (2022). 717–43.

[142] Shahid J Kashif A Shahid MK. A comprehensive review of physical therapy interventions for stroke rehabilitation: impairment-based approaches and functional goals. Brain Sci. (2023) 13:717. doi: 10.3390/brainsci13050717, PMID: 37239189 PMC10216461

[143] Hatem SM Saussez G Della Faille M Prist V Zhang X Dispa D. Rehabilitation of motor function after stroke: a multiple systematic review focused on techniques to stimulate upper extremity recovery. Front Hum Neurosci. (2016) 10:442. doi: 10.3389/fnhum.2016.00442 27679565 PMC5020059

[144] Cabanas-Valdés R Bagur-Calafat C Girabent-Farrés M Caballero-Gómez FM Hernández-Valiño M Urrútia CG. The effect of additional core stability exercises on improving dynamic sitting balance and trunk control for subacute stroke patients: a randomized controlled trial. Clin Rehabil. (2016) 30:1024–33. doi: 10.1177/0269215515609414, PMID: 26451007

[145] Joshua AM Karthikbabu S. Therapeutic approaches In: Joshua AM, editor. Physiotherapy for adult neurological conditions. Singapore: Springer (2022). 31–183.

[146] Gorst T Lyddon A Marsden J Paton J Morrison SC Cramp M. Foot and ankle impairments affect balance and mobility in stroke (FAiMiS): the views and experiences of people with stroke. Disabil Rehabil. (2016) 38:589–96. doi: 10.3109/09638288.2015.1052888, PMID: 26056857

[147] Willems EM Vermeulen J van Haastregt JC Zijlstra GR. Technologies to improve the participation of stroke patients in their home environment. Disabil Rehabil. (2022) 44:7116–26. doi: 10.1080/09638288.2021.1983041 34607474

[148] Chen Y Abel KT Janecek JT Chen Y Zheng K Cramer SC. Home-based technologies for stroke rehabilitation: a systematic review. Int J Med Inform. (2019) 123:11–22. doi: 10.1016/j.ijmedinf.2018.12.001, PMID: 30654899 PMC6814146

[149] Walder K Molineux M. Re-establishing an occupational identity after stroke—a theoretical model based on survivor experience. Br J Occup Ther. (2017) 80:620–30. doi: 10.1177/0308022617722711

[150] Hoffmann M Gustafsson L Di Tommaso A. Exploring stroke survivors’ experiences and understandings of occupational therapy. Scand J Occup Ther. (2022) 29:165–74. doi: 10.1080/11038128.2020.1831060, PMID: 33054465

[151] Wong MN-K Cheung MK-T Ng Y-M Yuan H-L Lam BY-H Fu SN. International classification of functioning, disability, and health-based rehabilitation program promotes activity and participation of post-stroke patients. Front Neurol. (2023) 14:1235500. doi: 10.3389/fneur.2023.1235500 38020626 PMC10657202

[152] Waddell KJ Birkenmeier RL Bland MD Lang CE. An exploratory analysis of the self-reported goals of individuals with chronic upper-extremity paresis following stroke. Disabil Rehabil. (2016) 38:853–7. doi: 10.3109/09638288.2015.1062926, PMID: 26146964 PMC4809414

[153] Hildebrand MW Geller D Proffitt R. Occupational therapy practice guidelines for adults with stroke. Am J Occup Ther. (2023) 77:7705397010. doi: 10.5014/ajot.2023.077501 37862268

[154] Richards LG Latham NK Jette DU Rosenberg L Smout RJ DeJong G. Characterizing occupational therapy practice in stroke rehabilitation. Arch Phys Med Rehabil. (2005) 86:51–60. doi: 10.1016/j.apmr.2005.08.127 16373140

[155] Broeren J Sunnerhagen KS Rydmark M. Haptic virtual rehabilitation in stroke: transferring research into clinical practice. Phys Ther Rev. (2009) 14:322–35. doi: 10.1179/108331909X12488667117212

[156] Burns SP Schwartz JK Scott SL Devos H Kovic M Hong I. Interdisciplinary approaches to facilitate return to driving and return to work in mild stroke: a position paper. Arch Phys Med Rehabil. (2018) 99:2378–88. doi: 10.1016/j.apmr.2018.01.032, PMID: 29518375

[157] Murray A Watter K McLennan V Vogler J Nielsen M Jeffery S. Identifying models, processes, and components of vocational rehabilitation following acquired brain injury: a systematic scoping review. Disabil Rehabil. (2022) 44:7641–54. doi: 10.1080/09638288.2021.1980622, PMID: 34606380

[158] Parisi A Bellinzona F Di Lernia D Repetto C De Gaspari S Brizzi G. Efficacy of multisensory Technology in Post-Stroke Cognitive Rehabilitation: a systematic review. J Clin Med. (2022) 11:6324. doi: 10.3390/jcm11216324, PMID: 36362551 PMC9656411

[159] Govender P Kalra L. Benefits of occupational therapy in stroke rehabilitation. Expert Rev Neurother. (2007) 7:1013–9. doi: 10.1586/14737175.7.8.1013 17678496

[160] Hatfield B Millet D Coles J Gassaway J Conroy B Smout RJ. Characterizing speech and language pathology outcomes in stroke rehabilitation. Arch Phys Med Rehabil. (2005) 86:61–72. doi: 10.1016/j.apmr.2005.08.111, PMID: 16373141

[161] Schwab SM Dugan S Riley MA. Reciprocal influence of mobility and speech-language: advancing physical therapy and speech therapy cotreatment and collaboration for adults with neurological conditions. Phys Ther. (2021) 101:pzab196. doi: 10.1093/ptj/pzab196, PMID: 34403483 PMC8801003

[162] Marzouqah R Huynh A Chen JL Boulos MI Yunusova Y. The role of oral and pharyngeal motor exercises in post-stroke recovery: a scoping review. Clin Rehabil. (2023) 37:620–35. doi: 10.1177/02692155221141395, PMID: 36426582 PMC10041576

[163] Sheppard SM Sebastian R. Diagnosing and managing post-stroke aphasia. Expert Rev Neurother. (2021) 21:221–34. doi: 10.1080/14737175.2020.1855976, PMID: 33231117 PMC7880889

[164] Ramsey A Blake ML. Speech-language pathology practices for adults with right hemisphere stroke: what are we missing? Am J Speech Lang Pathol. (2020) 29:741–59. doi: 10.1044/2020_AJSLP-19-00082, PMID: 32330389

[165] Speyer R Baijens L Heijnen M Zwijnenberg I. Effects of therapy in oropharyngeal dysphagia by speech and language therapists: a systematic review. Dysphagia. (2010) 25:40–65. doi: 10.1007/s00455-009-9239-7, PMID: 19760458 PMC2846331

[166] Kedlaya D Brandstater ME. Swallowing, nutrition, and hydration during acute stroke care. Top Stroke Rehabil. (2002) 9:23–38. doi: 10.1310/WEHA-ALJX-9N2X-0VMU, PMID: 14523715

[167] Moffatt K Pourshahid G Baecker RM. Augmentative and alternative communication devices for aphasia: the emerging role of “smart” mobile devices. Univ Access Inf Soc. (2017) 16:115–28. doi: 10.1007/s10209-015-0428-x

[168] Chang HF Power E O’Halloran R Foster A. Stroke communication partner training: a national survey of 122 clinicians on current practice patterns and perceived implementation barriers and facilitators. Int J Lang Commun Disord. (2018) 53:1094–109. doi: 10.1111/1460-6984.12421, PMID: 30151877

[169] Chiaramonte R Vecchio M. Dysarthria and stroke. The effectiveness of speech rehabilitation. A systematic review and meta-analysis of the studies. Eur J Phys Rehabil Med. (2021) 57:24–43. doi: 10.23736/S1973-9087.20.06242-5, PMID: 32519528

[170] Barman A Chatterjee A Bhide R. Cognitive impairment and rehabilitation strategies after traumatic brain injury. Indian J Psychol Med. (2016) 38:172–81. doi: 10.4103/0253-7176.183086, PMID: 27335510 PMC4904751

[171] Evans JJ. Memory dysfunction In: Textbook of neural repair and rehabilitation (2014). 478–88.

[172] de Lima MFR Cavendish BA de Deus JS Buratto LG. Retrieval practice in memory-and language-impaired populations: a systematic review. Arch Clin Neuropsychol. (2020) 35:1078–93. doi: 10.1093/arclin/acaa035 32514557

[173] Hyndman D Ashburn A. People with stroke living in the community: attention deficits, balance, ADL ability and falls. Disabil Rehabil. (2003) 25:817–22. doi: 10.1080/0963828031000122221, PMID: 12851091

[174] McCulloch K. Attention and dual-task conditions: physical therapy implications for individuals with acquired brain injury. J Neurol Phys Ther. (2007) 31:104–18. doi: 10.1097/NPT.0b013e31814a6493, PMID: 18025956

[175] Taylor GH Broomfield NM. Cognitive assessment and rehabilitation pathway for stroke (CARPS). Top Stroke Rehabil. (2013) 20:270–82. doi: 10.1310/tsr2003-270, PMID: 23841975

[176] Hoffmann T McKenna K. Analysis of stroke patients’ and carers’ reading ability and the content and design of written materials: recommendations for improving written stroke information. Patient Educ Couns. (2006) 60:286–93. doi: 10.1016/j.pec.2005.06.020, PMID: 16098708

[177] McEwen S Polatajko H Baum C Rios J Cirone D Doherty M. Combined cognitive-strategy and task-specific training improve transfer to untrained activities in subacute stroke: an exploratory randomized controlled trial. Neurorehabil Neural Repair. (2015) 29:526–36. doi: 10.1177/1545968314558602, PMID: 25416738 PMC4440855

[178] Cicerone KD Dahlberg C Kalmar K Langenbahn DM Malec JF Bergquist TF. Evidence-based cognitive rehabilitation: recommendations for clinical practice. Arch Phys Med Rehabil. (2000) 81:1596–615. doi: 10.1053/apmr.2000.19240 11128897

[179] Pang MYC Yang L Ouyang H Lam FMH Huang M Jehu DA. Dual-task exercise reduces cognitive-motor interference in walking and falls after stroke: a randomized controlled study. Stroke. (2018) 49:2990–8. doi: 10.1161/STROKEAHA.118.022157, PMID: 30571419

[180] Mancioppi G Fiorini L Rovini E Cavallo F. The use of motor and cognitive dual-task quantitative assessment on subjects with mild cognitive impairment: a systematic review. Mech Ageing Dev. (2021) 193:111393. doi: 10.1016/j.mad.2020.111393, PMID: 33188785

[181] Ogbeiwi O. General concepts of goals and goal-setting in healthcare: a narrative review. J Manag Organ. (2021) 27:324–41. doi: 10.1017/jmo.2018.11

[182] Hillig T Ma H Dorsch S. Goal-oriented instructions increase the intensity of practice in stroke rehabilitation compared with non-specific instructions: a within-participant, repeated measures experimental study. J Physiother. (2019) 65:95–8. doi: 10.1016/j.jphys.2019.02.007, PMID: 30910568

[183] Andringa A van de Port I van Wegen E Ket J Meskers C Kwakkel G. Effectiveness of botulinum toxin treatment for upper limb spasticity poststroke over different ICF domains: a systematic review and meta-analysis. Arch Phys Med Rehabil. (2019) 100:1703–25. doi: 10.1016/j.apmr.2019.01.016 30796921

[184] Suputtitada A Chatromyen S Chen CP Simpson DM. Best practice guidelines for the management of patients with post-stroke spasticity: a modified scoping review. Toxins. (2024) 16:98. doi: 10.3390/toxins16020098, PMID: 38393176 PMC10892074

[185] Mills PB Finlayson H Sudol M O’Connor R. Systematic review of adjunct therapies to improve outcomes following botulinum toxin injection for treatment of limb spasticity. Clin Rehabil. (2016) 30:537–48. doi: 10.1177/0269215515593783, PMID: 26198891

[186] Thibaut A Chatelle C Ziegler E Bruno M-A Laureys S Gosseries O. Spasticity after stroke: physiology, assessment and treatment. Brain Inj. (2013) 27:1093–105. doi: 10.3109/02699052.2013.804202 23885710

[187] Baricich A Picelli A Santamato A Carda S de Sire A Smania N. Safety profile of high-dose botulinum toxin type a in post-stroke spasticity treatment. Clin Drug Investig. (2018) 38:991–1000. doi: 10.1007/s40261-018-0701-x, PMID: 30209743

[188] Santamato A Panza F. Benefits and risks of non-approved injection regimens for botulinum toxins in spasticity. Drugs. (2017) 77:1413–22. doi: 10.1007/s40265-017-0786-1, PMID: 28726023

[189] Facciorusso S Spina S Picelli A Baricich A Francisco GE Molteni F. The role of botulinum toxin type-a in spasticity: research trends from a bibliometric analysis. Toxins. (2024) 16:184. doi: 10.3390/toxins16040184, PMID: 38668609 PMC11053519

[190] Santamato A Micello MF Ranieri M Valeno G Albano A Baricich A. Employment of higher doses of botulinum toxin type a to reduce spasticity after stroke. J Neurol Sci. (2015) 350:1–6. doi: 10.1016/j.jns.2015.01.033 25684341

[191] Engineer ND Kimberley TJ Prudente CN Dawson J Tarver WB Hays SA. Targeted vagus nerve stimulation for rehabilitation after stroke. Front Neurosci. (2019) 13:280. doi: 10.3389/fnins.2019.00280, PMID: 30983963 PMC6449801

[192] Khalid S Alnajjar F Gochoo M Renawi A Shimoda S. Robotic assistive and rehabilitation devices leading to motor recovery in upper limb: a systematic review. Disabil Rehabil Assist Technol. (2023) 18:658–72. doi: 10.1080/17483107.2021.1906960, PMID: 33861684

[193] Molteni F Gasperini G Cannaviello G Guanziroli E. Exoskeleton and end-effector robots for upper and lower limbs rehabilitation: narrative review. PM R. (2018) 10:S174–88. doi: 10.1016/j.pmrj.2018.06.005 30269804

[194] Louie DR Eng JJ. Powered robotic exoskeletons in post-stroke rehabilitation of gait: a scoping review. J Neuroeng Rehabil. (2016) 13:53. doi: 10.1186/s12984-016-0162-5 27278136 PMC4898381

[195] Spencer J Wolf SL Kesar TM. Biofeedback for post-stroke gait retraining: a review of current evidence and future research directions in the context of emerging technologies. Front Neurol. (2021) 12:637199. doi: 10.3389/fneur.2021.637199, PMID: 33859607 PMC8042129

[196] Hobbs B Artemiadis P. A review of robot-assisted lower-limb stroke therapy: unexplored paths and future directions in gait rehabilitation. Front Neurorobot. (2020) 14:19. doi: 10.3389/fnbot.2020.00019, PMID: 32351377 PMC7174593

[197] Pilla A Trigili E McKinney Z Fanciullacci C Malasoma C Posteraro F. Robotic rehabilitation and multimodal instrumented assessment of post-stroke elbow motor functions—a randomized controlled trial protocol. Front Neurol. (2020) 11:587293. doi: 10.3389/fneur.2020.587293 33193052 PMC7643017

[198] Cherry COB Chumbler NR Richards K Huff A Wu D Tilghman LM. Expanding stroke telerehabilitation services to rural veterans: a qualitative study on patient experiences using the robotic stroke therapy delivery and monitoring system program. Disabil Rehabil Assist Technol. (2017) 12:21–7. doi: 10.3109/17483107.2015.1061613 26135221

[199] Das A Day TW Kulkarni V Buchanan A Cottrell K John NW. Towards intelligent extended reality in stroke rehabilitation: application of machine learning and artificial intelligence in rehabilitation In: Augmenting neurological disorder prediction and rehabilitation using artificial intelligence: Elsevier (2022). 309–29.

[200] Tuah NM Ahmedy F Gani A Yong LN. A survey on gamification for health rehabilitation care: applications, opportunities, and open challenges. Information. (2021) 12:91. doi: 10.3390/info12020091

[201] Weber LM Stein J. The use of robots in stroke rehabilitation: a narrative review. NeuroRehabilitation. (2018) 43:99–110. 30056437 10.3233/NRE-172408

[202] Takebayashi T Takahashi K Okita Y Kubo H Hachisuka K Domen K. Impact of the robotic-assistance level on upper extremity function in stroke patients receiving adjunct robotic rehabilitation: sub-analysis of a randomized clinical trial. J Neuroeng Rehabil. (2022) 19:25. doi: 10.1186/s12984-022-00986-9, PMID: 35216603 PMC8881821

[203] Secoli R Milot M-H Rosati G Reinkensmeyer DJ. Effect of visual distraction and auditory feedback on patient effort during robot-assisted movement training after stroke. J Neuroeng Rehabil. (2011) 8:21. doi: 10.1186/1743-0003-8-21, PMID: 21513561 PMC3104373

[204] Turchetti G Vitiello N Trieste L Romiti S Geisler E Micera S. Why effectiveness of robot-mediated neurorehabilitation does not necessarily influence its adoption. IEEE Rev Biomed Eng. (2014) 7:143–53. doi: 10.1109/RBME.2014.2300234, PMID: 24803207

[205] Gorman C Gustafsson L. The use of augmented reality for rehabilitation after stroke: a narrative review. Disabil Rehabil Assist Technol. (2022) 17:409–17. doi: 10.1080/17483107.2020.1791264, PMID: 32663112

[206] Kim W-S Cho S Ku J Kim Y Lee K Hwang H-J. Clinical application of virtual reality for upper limb motor rehabilitation in stroke: review of technologies and clinical evidence. J Clin Med. (2020) 9:3369. doi: 10.3390/jcm9103369, PMID: 33096678 PMC7590210

[207] Sun X Ding J Dong Y Ma X Wang R Jin K. A survey of technologies facilitating home and community-based stroke rehabilitation. Int J Hum-Comput Interact. (2023) 39:1016–42. doi: 10.1080/10447318.2022.2050545

[208] Patil V Narayan J Sandhu K Dwivedy SK. Integration of virtual reality and augmented reality in physical rehabilitation: a state-of-the-art review In: Revolutions in product design for healthcare: advances in product design and design methods for healthcare. Singapore: Springer (2022). 177–205.

[209] Kiper P Szczudlik A Agostini M Opara J Nowobilski R Ventura L. Virtual reality for upper limb rehabilitation in subacute and chronic stroke: a randomized controlled trial. Arch Phys Med Rehabil. (2018) 99:834–842.e4. doi: 10.1016/j.apmr.2018.01.023, PMID: 29453980

[210] Draaisma LR Wessel MJ Hummel FC. Neurotechnologies as tools for cognitive rehabilitation in stroke patients. Expert Rev Neurother. (2020) 20:1249–61. doi: 10.1080/14737175.2020.1820324, PMID: 32887528

[211] Maggio MG Latella D Maresca G Sciarrone F Manuli A Naro A. Virtual reality and cognitive rehabilitation in people with stroke: an overview. J Neurosci Nurs. (2019) 51:101–5. doi: 10.1097/JNN.0000000000000423, PMID: 30649091

[212] Villeneuve M Ogourtsova T Deblock-Bellamy A Blanchette A Bühler MA Fung J. Development of a virtual reality-based intervention for community walking post stroke: an integrated knowledge translation approach. Disabil Rehabil. (2023):1–11. doi: 10.1080/09638288.2023.2277397 37921690

[213] Faria AL Latorre J Cameirão MS Bermúdez I Badia S Llorens R. Ecologically valid virtual reality-based technologies for assessment and rehabilitation of acquired brain injury: a systematic review. Front Psychol. (2023) 14:1233346. doi: 10.3389/fpsyg.2023.1233346 37711328 PMC10497882

[214] Montalbán MA Arrogante O. Rehabilitation through virtual reality therapy after a stroke: a literature review In: Revista Científica de la Sociedad de Enfermería Neurológica (English ed.) (2020). 19–27.

[215] Glegg SMN Levac DE. Barriers, facilitators and interventions to support virtual reality implementation in rehabilitation: a scoping review. PM R. (2018) 10:1237–51.e1. doi: 10.1016/j.pmrj.2018.07.004, PMID: 30503231 PMC6752033

[216] Charles D Holmes D Charles T McDonough S. Virtual reality design for stroke rehabilitation In: Biomedical visualisation. Advances in experimental medicine and biology. Cham: Springer (2020). 53–87. 10.1007/978-3-030-37639-0_4 32488636

[217] Zuki FSM Sulaiman S Rambli DRA Merienne F Saad MNM. (2021). Sensory feedback and interactivity: Enhancing motivation and engagement for VR stroke rehabilitation. 2021 International Conference on Computer & Information Sciences (ICCOINS): IEEE.

[218] Hui J Lioumis P Blumberger DM Daskalakis ZJ. Non-invasive central neuromodulation with transcranial magnetic stimulation In: Stereotactic and functional neurosurgery: principles and applications. Cham: Springer (2020). 205–22.

[219] Fisicaro F Lanza G Grasso AA Pennisi G Bella R Paulus W. Repetitive transcranial magnetic stimulation in stroke rehabilitation: review of the current evidence and pitfalls. Ther Adv Neurol Disord. (2019) 12:175628641987831. doi: 10.1177/1756286419878317 PMC6763938 31598137

[220] Starosta M Cichoń N Saluk-Bijak J Miller E. Benefits from repetitive transcranial magnetic stimulation in post-stroke rehabilitation. J Clin Med. (2022) 11:2149. doi: 10.3390/jcm11082149, PMID: 35456245 PMC9030945

[221] Liepert J Bauder H Miltner WH Taub E Weiller C. Treatment-induced cortical reorganization after stroke in humans. Stroke. (2000) 31:1210–6. doi: 10.1161/01.STR.31.6.1210, PMID: 10835434

[222] Lai C-J Wang C-P Tsai P-Y Chan R-C Lin S-H Lin F-G. Corticospinal integrity and motor impairment predict outcomes after excitatory repetitive transcranial magnetic stimulation: a preliminary study. Arch Phys Med Rehabil. (2015) 96:69–75. doi: 10.1016/j.apmr.2014.08.014, PMID: 25218256

[223] Auriat AM Neva JL Peters S Ferris JK Boyd LA. A review of transcranial magnetic stimulation and multimodal neuroimaging to characterize post-stroke neuroplasticity. Front Neurol. (2015) 6:226. doi: 10.3389/fneur.2015.00226 26579069 PMC4625082

[224] Giordano J Bikson M Kappenman ES Clark VP Coslett HB Hamblin MR. Mechanisms and effects of transcranial direct current stimulation. Dose Response. (2017) 15:1559325816685467. doi: 10.1177/1559325816685467, PMID: 28210202 PMC5302097

[225] Santos Ferreira I Teixeira Costa B Lima Ramos C Lucena P Thibaut A Fregni F. Searching for the optimal tDCS target for motor rehabilitation. J Neuroeng Rehabil. (2019) 16:90. doi: 10.1186/s12984-019-0561-5 31315679 PMC6637619

[226] Lefebvre S Liew S-L. Anatomical parameters of tDCS to modulate the motor system after stroke: a review. Front Neurol. (2017) 8:29. doi: 10.3389/fneur.2017.00029 28232816 PMC5298973

[227] Otal B Dutta A Foerster Á Ripolles O Kuceyeski A Miranda PC. Opportunities for guided multichannel non-invasive transcranial current stimulation in poststroke rehabilitation. Front Neurol. (2016) 7:21. doi: 10.3389/fneur.2016.00021 26941708 PMC4764713

[228] Guimarães AN Porto AB Marcori AJ Lage GM Altimari LR Okazaki VHA. Motor learning and tDCS: a systematic review on the dependency of the stimulation effect on motor task characteristics or tDCS assembly specifications. Neuropsychologia. (2023) 179:108463. doi: 10.1016/j.neuropsychologia.2022.108463, PMID: 36567006

[229] Olgiati E Malhotra PA. Using non-invasive transcranial direct current stimulation for neglect and associated attentional deficits following stroke. Neuropsychol Rehabil. (2022) 32:735–66. doi: 10.1080/09602011.2020.1805335, PMID: 32892712

[230] Takeda K Tanino G Miyasaka H. Review of devices used in neuromuscular electrical stimulation for stroke rehabilitation In: Medical devices: evidence and research: Taylor & Francis (2017). 207–13. 10.2147/MDER.S123464 PMC5576704 28883745

[231] Chae J Yu DT. Neuromuscular electrical stimulation for motor restoration in hemiparesis. Top Stroke Rehabil. (2002) 8:24–39. doi: 10.1310/REXB-AKV9-2XBE-U5QA 14523728

[232] Sabut SK Sikdar C Mondal R Kumar R Mahadevappa M. Restoration of gait and motor recovery by functional electrical stimulation therapy in persons with stroke. Disabil Rehabil. (2010) 32:1594–603. doi: 10.3109/09638281003599596, PMID: 20210592

[233] Nagai MK Marquez-Chin C Popovic MR. Why is functional electrical stimulation therapy capable of restoring motor function following severe injury to the central nervous system? In: Translational neuroscience: fundamental approaches for neurological disorders. Boston, MA: Springer (2016). 479–98.

[234] Cheung VC Niu CM Li S Xie Q Lan N. A novel FES strategy for poststroke rehabilitation based on the natural organization of neuromuscular control. IEEE Rev Biomed Eng. (2018) 12:154–67. doi: 10.1109/RBME.2018.2874132 30307876

[235] Tepavac D Schwirtlich L. Detection and prediction of FES-induced fatigue. J Electromyogr Kinesiol. (1997) 7:39–50. doi: 10.1016/S1050-6411(96)00008-9, PMID: 20719690

[236] Niu CM Bao Y Zhuang C Li S Wang T Cui L. Synergy-based FES for post-stroke rehabilitation of upper-limb motor functions. IEEE Trans Neural Syst Rehabil Eng. (2019) 27:256–64. doi: 10.1109/TNSRE.2019.2891004, PMID: 30763238

[237] Lyons G Wilcox D Lyons D Hilton D. (2000). Evaluation of a drop foot stimulator FES intensity envelope matched to tibialis anterior muscle activity during walking. Proceedings of Fifth Annual Conference of the International Functional Electrical Stimulation Society: IFESS. Aalborg.

[238] Bala S Vishnu VY Joshi D. Muscle synergy-based functional electrical stimulation reduces muscular fatigue in post-stroke patients: a systematic comparison. IEEE Trans Neural Syst Rehabil Eng. (2023) 31:2858–71. doi: 10.1109/TNSRE.2023.3290293, PMID: 37379181

[239] Hebert D Lindsay MP McIntyre A Kirton A Rumney PG Bagg S. Canadian stroke best practice recommendations: stroke rehabilitation practice guidelines, update 2015. Int J Stroke. (2016) 11:459–84. doi: 10.1177/1747493016643553, PMID: 27079654

[240] Paul D Mukherjee M Bakshi A. A review of brain-computer interface In: Advances in medical physics and healthcare engineering. Singapore: Springer (2020). 507–31.

[241] Chaudhary U Birbaumer N Ramos-Murguialday A. Brain-computer interfaces for communication and rehabilitation. Nat Rev Neurol. (2016) 12:513–25. doi: 10.1038/nrneurol.2016.113 27539560

[242] Mane R Ang KK Guan C. Brain-computer interface for stroke rehabilitation In: Handbook of neuroengineering. Singapore: Springer (2023). 1285–315.

[243] He B Yuan H Meng J Gao S. Brain-computer interfaces In: Neural engineering. Cham: Springer (2020). 131–83.

[244] Ramadan RA Vasilakos AV. Brain computer interface: control signals review. Neurocomputing. (2017) 223:26–44. doi: 10.1016/j.neucom.2016.10.024

[245] Daly JJ Wolpaw JR. Brain-computer interfaces in neurological rehabilitation. Lancet Neurol. (2008) 7:1032–43. doi: 10.1016/S1474-4422(08)70223-0 18835541

[246] Remsik A Young B Vermilyea R Kiekhoefer L Abrams J Evander Elmore S. A review of the progression and future implications of brain-computer interface therapies for restoration of distal upper extremity motor function after stroke. Expert Rev Med Devices. (2016) 13:445–54. doi: 10.1080/17434440.2016.1174572, PMID: 27112213 PMC5131699

[247] Bernhardt J Hayward KS Dancause N Lannin NA Ward NS Nudo RJ. A stroke recovery trial development framework: consensus-based core recommendations from the second stroke recovery and rehabilitation roundtable. Neurorehabil Neural Repair. (2019) 33:959–69. doi: 10.1177/1545968319888642 31674274

[248] Korner-Bitensky N Barrett-Bernstein S Bibas G Poulin V. National survey of Canadian occupational therapists’ assessment and treatment of cognitive impairment post-stroke. Aust Occup Ther J. (2011) 58:241–50. doi: 10.1111/j.1440-1630.2011.00943.x, PMID: 21770959

[249] Seabury S Bognar K Xu Y Huber C Commerford SR Tayama D. Regional disparities in the quality of stroke care. Am J Emerg Med. (2017) 35:1234–9. doi: 10.1016/j.ajem.2017.03.046 28431874

[250] Longley V Peters S Swarbrick C Bowen A. What factors affect clinical decision-making about access to stroke rehabilitation? A systematic review. Clin Rehabil. (2019) 33:304–16. doi: 10.1177/0269215518808000, PMID: 30370792 PMC6348456

[251] Palozzi G Schettini I Chirico A. Enhancing the sustainable goal of access to healthcare: findings from a literature review on telemedicine employment in rural areas. Sustainability. (2020) 12:3318. doi: 10.3390/su12083318

[252] Alhasani R Radman D Auger C Lamontagne A Ahmed S. Perspectives of clinicians and survivors on the continuity of service provision during rehabilitation after acquired brain injury. PLoS One. (2023) 18:e0284375. doi: 10.1371/journal.pone.0284375 37043494 PMC10096466

[253] Miller CM Behrouz R. Impact of infection on stroke morbidity and outcomes. Curr Neurol Neurosci Rep. (2016) 16:83. doi: 10.1007/s11910-016-0679-9 27485944

[254] Poisson SN Johnston SC Josephson SA. Urinary tract infections complicating stroke: mechanisms, consequences, and possible solutions. Stroke. (2010) 41:e180–4. doi: 10.1161/STROKEAHA.109.576413, PMID: 20167905

[255] Katzan IL Cebul RD Husak S Dawson N Baker D. The effect of pneumonia on mortality among patients hospitalized for acute stroke. Neurology. (2003) 60:620–5. doi: 10.1212/01.WNL.0000046586.38284.60 12601102

[256] Langhorne P Stott D Robertson L Mac Donald J Jones L McAlpine C. Medical complications after stroke: a multicenter study. Stroke. (2000) 31:1223–9. doi: 10.1161/01.STR.31.6.1223 10835436

[257] Westendorp WF Nederkoorn PJ Vermeij J-D Dijkgraaf MG van de Beek D. Post-stroke infection: a systematic review and meta-analysis. BMC Neurol. (2011) 11:110. doi: 10.1186/1471-2377-11-110 21933425 PMC3185266

[258] Finlayson O Kapral M Hall R Asllani E Selchen D Saposnik G. Risk factors, inpatient care, and outcomes of pneumonia after ischemic stroke. Neurology. (2011) 77:1338–45. doi: 10.1212/WNL.0b013e31823152b1 21940613

[259] Sumner J Lim HW Chong LS Bundele A Mukhopadhyay A Kayambu G. Artificial intelligence in physical rehabilitation: a systematic review. Artif Intell Med. (2023) 146:102693. doi: 10.1016/j.artmed.2023.102693, PMID: 38042593

[260] Adans-Dester CP Lang CE Reinkensmeyer DJ Bonato P. Wearable sensors for stroke rehabilitation In: Neurorehabilitation technology: Springer International Publishing (2022). 467–507.

[261] Teasell R Salbach NM Foley N Mountain A Cameron JI Jong A. Canadian stroke best practice recommendations: rehabilitation, recovery, and community participation following stroke. Part one: rehabilitation and recovery following stroke; update 2019. Int J Stroke. (2020) 15:763–88. doi: 10.1177/1747493019897843, PMID: 31983296

[262] Clarke DJ Forster A. Improving post-stroke recovery: the role of the multidisciplinary health care team. J Multidiscip Healthc. (2015) 8:433–42. doi: 10.2147/JMDH.S68764, PMID: 26445548 PMC4590569

[263] Tosto-Mancuso J Tabacof L Herrera JE Breyman E Dewil S Cortes M. Gamified neurorehabilitation strategies for post-stroke motor recovery: challenges and advantages. Curr Neurol Neurosci Rep. (2022) 22:183–95. doi: 10.1007/s11910-022-01181-y, PMID: 35278172 PMC8917333

[264] Aderinto N Abdul Basit MO Olatunji G Adejumo T. Exploring the transformative influence of neuroplasticity on stroke rehabilitation: a narrative review of current evidence. Ann Med Surg. (2023) 85:4425–32. doi: 10.1097/MS9.0000000000001137, PMID: 37663728 PMC10473303

[265] Baniqued PDE Stanyer EC Awais M Alazmani A Jackson AE Mon-Williams MA. Brain-computer interface robotics for hand rehabilitation after stroke: a systematic review. J Neuroeng Rehabil. (2021) 18:15. doi: 10.1186/s12984-021-00820-8 33485365 PMC7825186

